# Ribosome Traffic on mRNAs Maps to Gene Ontology: Genome-wide Quantification of Translation Initiation Rates and Polysome Size Regulation

**DOI:** 10.1371/journal.pcbi.1002866

**Published:** 2013-01-31

**Authors:** Luca Ciandrini, Ian Stansfield, M. Carmen Romano

**Affiliations:** 1 SUPA, Institute for Complex Systems and Mathematical Biology, King's College, University of Aberdeen, Aberdeen, United Kingdom; 2 Institute of Medical Sciences, Foresterhill, University of Aberdeen, Aberdeen, United Kingdom; University of Chicago, United States of America

## Abstract

To understand the complex relationship governing transcript abundance and the level of the encoded protein, we integrate genome-wide experimental data of ribosomal density on mRNAs with a novel stochastic model describing ribosome traffic dynamics during translation elongation. This analysis reveals that codon arrangement, rather than simply codon bias, has a key role in determining translational efficiency. It also reveals that translation output is governed both by initiation efficiency and elongation dynamics. By integrating genome-wide experimental data sets with simulation of ribosome traffic on all *Saccharomyces cerevisiae* ORFs, mRNA-specific translation initiation rates are for the first time estimated across the entire transcriptome. Our analysis identifies different classes of mRNAs characterised by their initiation rates, their ribosome traffic dynamics, and by their response to ribosome availability. Strikingly, this classification based on translational dynamics maps onto key gene ontological classifications, revealing evolutionary optimisation of translation responses to be strongly influenced by gene function.

## Author Summary

Gene expression regulation is central to all living systems. Here we introduce a new framework and methodology to study the last stage of protein production in cells, where the genetic information encoded in the mRNAs is translated from the language of nucleotides into functional proteins. The process, on each mRNA, is carried out concurrently by several ribosomes; like cars on a small countryside road, they cannot overtake each other, and can form queues. By integrating experimental data with genome-wide simulations of our model, we analyse ribosome traffic across the entire *Saccharomyces cerevisiae* genome, and for the first time estimate mRNA-specific translation initiation rates for each transcript. Crucially, we identify different classes of mRNAs characterised by different ribosome traffic dynamics. Remarkably, this classification based on translational dynamics, and the evaluation of mRNA-specific initiation rates, map onto key gene ontological classifications, revealing evolutionary optimisation of translation responses to be strongly influenced by gene function.

## Introduction

The expression of genes can be considered as a two-stage process, beginning with transcription and the production of an mRNA, followed by translation of that mRNA into protein by the cell's ribosome population. Gene expression must be tightly regulated to control protein composition, enabling the cell to rapidly respond to a wide range of environmental conditions. For this reason, cells exert fine control over gene expression, both at the transcriptional [1], [2] and post-transcriptional level [3]–[6].

One key mechanism of post-transcriptional control of gene expression is translational regulation. The process of translation can be divided in three main phases, namely initiation, elongation and termination. Whereas termination is generally believed to be a fast process and therefore not limiting for translation [7], the respective contributions of initiation and elongation to translational regulation are still under debate [8].

On one hand, the translation initiation rate, or the rate at which ribosomes access the 5′ untranslated region (5′ UTR) and start translating the ORF, is regulated in part by formation of secondary structures in the 5′ leader [9], [10]. The presence of secondary structures inhibits the ability of an mRNA to sequester ribosomes, thereby lowering the effective translation initiation rate. The 5′ leader composition is characteristic of each mRNA, resulting in a heterogeneity of the ribosome recruitment process among the transcripts [11], [12]. Despite the importance of this process in gene expression regulation, there are currently no estimates of *in vivo*, mRNA-specific translation initiation rates based on refined traffic models, and how they regulate genome-wide patterns of protein expression. On the other hand, there is increasing evidence that translation elongation itself controls gene expression, being regulated by the rate of supply of tRNAs, particularly in microorganisms with codon biased genomes. Within families of isoacceptor tRNAs, members are not all present at the same concentration in the cell, leading to variation in delivery times, and the introduction of stochastic pauses [13]. Such pauses control ribosome transit, regulating ribosome queue formation. There is evidence that a ramp of slow codons near the 5′ end of some open reading frames regulates the flow of ribosomes onto an mRNA [14], [15], and pausing during elongation on any mRNA will affect queue dynamics, and thus the flux (or current) of ribosomes along the mRNA. However, there is no knowledge of how, on a genome-wide scale, the dynamic flux of ribosomes along an mRNA might be crucial in regulating protein expression.

Here, we address these two problems: first, we estimate mRNA-specific *in vivo* translation initiation rates on a genome-wide basis by integrating a computational model of mRNA translation with experimental datasets of ribosome occupancy. Crucially, we show that translation initiation rates are correlated with gene function. Second, we show that the translation dynamics response of each mRNA is characteristic of its gene ontology, by elucidating how ribosome traffic, moving with variable speed across the codon field, responds to a range of initiation rates. We also show that codon arrangement rather than codon usage, clearly separates mRNAs into distinct classes typified by their responses to variations of the translation initiation rate. This suggests that not only codon usage but also codon arrangement is a selectable determinant of gene expression.

## Results

### The model

**Figure 1:**
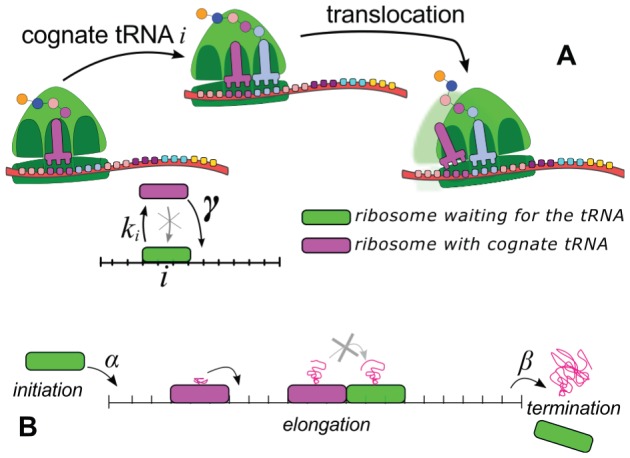
The model. Particles representing ribosomes move along a unidimensional lattice (the mRNA chain) in which each site represents a codon. For the sake of illustration, in the sketch a particle covers 3 codons, while in the model we considered particles occupying 9 codons [15]. (**A**) Schematic representation of ribosome dynamics: along the mRNA, ribosomes with the A site on codon 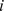 capture the cognate tRNA with a rate 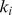, then keep it and advance with a rate 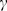, provided that the following codon is empty. (**B**) The entire translation process can be viewed as particles moving on a lattice. Ribosomes attempt to initiate the translation with a rate 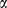. Then they move according to the dynamical rules introduced above and at the end of the lattice the ribosomes detach with a termination rate 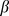. Particles can queue if the bottlenecks in the lattice cannot support the incoming flow.

Our model describes how ribosomes bind to the mRNA, move along it performing the translation, and dissociate from the mRNA at the stop codon, releasing the finished protein into the cytoplasm [16]. The mRNA is represented by a unidimensional lattice, with each site denoting a codon. Ribosomes are represented by particles occupying 9 codons [15] that attempt to bind the mRNA with a rate 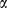, provided that the binding region is not obstructed by another ribosome. The particle on-rate 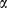 mimics the initiation of translation, in which several processes have been condensed into just one step. The factors influencing the initiation of translation, such as secondary structures in the 5′UTR, concentration of initiation factors and ribosome availability, are all included in this parameter and will be discussed below. Subsequently, ribosomes advance on the polynucleotide chain (elongation) following a two-state dynamics: (1) recognition of the cognate tRNA with rate 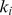 depending on the codon 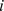, and (2) translocation towards the next codon with rate 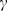 (see Figure 1). At the last codon, the ribosomes detach and release the protein with a rate  (termination).

The cognate tRNA-capture rates 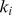 can be estimated from data on tRNA abundances, which are assumed to be proportional to their gene copy numbers [17], and by considering further corrections such as the wobble base pairing (see Supplementary Information, Text S1). Effects of competition for near-cognate and non-cognate tRNAS were found not to materially affect any of the conclusions of this study (see Supplementary Information, Text S1), and are therefore neglected. The translocation rate 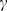 has been measured to be 35 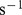 [18], and is codon independent. The termination rate is determined by the concentration of the release factors; the termination process is assumed to be fast, comparable to the translocation [7].

Moreover, the model takes into consideration steric interactions between ribosomes, so that even if a ribosome sitting on codon 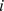 has already captured the cognate tRNA, it cannot translocate if the next codon is occupied by another ribosome. Hence, it is an exclusion process [19] exhibiting different regimes characterised by the flow of particles and by their density along the lattice. In particular, if the sequence contains slow sites, then queues of particles behind the slow sites or high density phases appear when the on-rate of particles is of the same magnitude as the bottleneck rate.

In contrast to commonly used exclusion models [14], [20], our model accounts for the processes involved in the mechano-chemical ribosome cycle, condensing them in two main steps: capture of the tRNA and translocation. It includes the crucial fact that ribosomes can capture a cognate tRNA while they wait for the next lattice position to become vacant. In contrast, ribosomes from simpler exclusion models unrealistically “lose” immediately the captured tRNA if they cannot move to the next codon. This is a key difference, which leads to different dynamics of ribosome traffic and transitions between traffic regimes [16]. This effect is further enhanced by the fact that the time scales related to the capture of the tRNA and translocation are strongly separated, with the translocation being much faster. Furthermore, the two-state ribosome reproduces the dwell-times observed in single-molecule experiments [21].

In summary, our model predicts the current of ribosomes 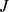 or translation rate, and the density 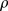 of ribosomes on a particular mRNA (number of ribosomes divided by the ORF length), taking as input the specific sequence of codons of the mRNA. Both the translation rate 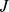 and the ribosome density 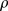 are predicted as a function of the translation initiation rate 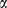, i.e. the rate at which ribosomes arrive at the start AUG codon; their functional dependence on the initiation rate thus varies from sequence to sequence as a consequence of different codon compositions and codon arrangements.

### Genome-wide prediction of translation initiation rates

The translation initiation rate 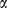, i.e. the rate at which ribosomes start translating the ORF, depends on many factors, such as the rate at which ribosomes attempt to bind the mRNA, the concentration of initiation factors and the presence of secondary structures in the 5′UTR region [9], [11], [12]. Despite the key role of this parameter, direct experimental evaluations are intractable, both *in vivo* and *in vitro*, with no direct measurements having been carried out to date.

Previous works, such as [20], could only estimate the translation initiation rate as the value that maximised the predicted correlation of the ribosome current with experimental data. Furthermore, 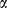 has usually been considered as a unique, fixed value (the same for each of the mRNAs), but it is well known that the translation initiation rate depends on several mRNA-specific factors, such as the structural properties of the mRNA leader region. Knowledge of mRNA-specific values of 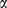, therefore, would provide important insight into control of gene expression at the level of translation. Siwiak and Zielenkiewicz [22] present specific initiation rates, however with a simple model that neglects ribosome kinetics and traffic (the comparison is discussed in the Supplementary Text S2). Here we present a novel approach to identify the initiation rate of each individual mRNA for the whole genome.

**Figure 2:**
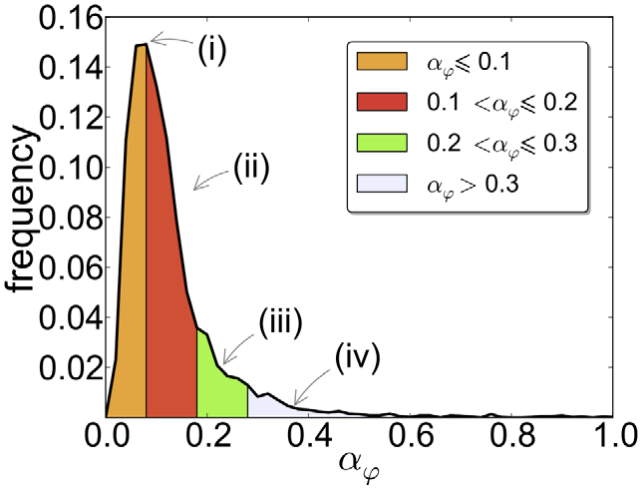
Distribution of the estimated initiation rates in *S. cerevisiae*. The mean initiation rate is 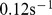 and the median is 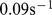. Most of the mRNAs have an estimated initiation rate 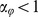 and therefore we show only this range.

We first apply our translation model to all mRNA sequences of *S. cerevisiae*. The model predicts the translation rate 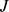 and the ribosome density 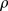 on each mRNA as a function of the translation initiation rate 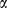. Then, by utilising genome-wide experimental data of ribosome density 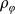 from [23] for yeast grown under non-stressed conditions, we identify the physiological translation initiation rate 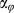 as the one which, when used in our simulations, replicates the experimentally observed density:
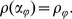
(1)This yields a value of the translation initiation rate for each mRNA 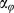 as shown by the genome-wide distribution in Figure 2. Using the genome-wide experimental data of ribosome density from Arava et al. [7] yields a very similar distribution of initiation rates (see Section 4 of Supplementary Text S1). The knowledge of this distribution reveals how translational regulation of gene expression works at the level of initiation by correlating the values of 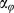 with the biological functions of the corresponding genes, encoded in their Gene Ontology (GO) annotations. In Figure 2 we split up this distribution in four parts, from small to high 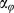 (i)–(iv). Strikingly, significantly enriched GO annotations are identifiable in each of the regions. Messenger RNAs with an initiation rate below 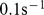 (region (i) of Figure 2) contain a highly disproportionate number of regulatory proteins and proteins linked to transcription from Pol II promoters, mainly located in the nucleus, chromosome, membrane or protein complexes. In the range of 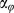 from 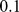 to 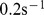 (region (ii) of Figure 2) we find other significantly over-represented terms such as cytoplasmic translation, ribosome biogenesis or oxoacid metabolic process, while genes with 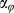 from 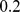 to 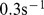 (region (iii) of Figure 2) are primarily constituents of ribosomes. Very large initiation rates (region (iv) of Figure 2) are characteristic of genes associated with the respiratory chain. However, most of genes falling in this region are not annotated (a complete list of 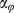 can be found in the Supplementary Table S1 and the details of the GO analysis, with the annotations found in each region and their enrichments, can be found in the Supplementary Table S2).

The assignment of initiation rates correlates with protein abundances typical of given GO categories: regulatory proteins are usually present at low levels. In contrast, proteins involved in translation, ribosome biogenesis and metabolic processes are abundant. This result is a signature of the divergent translational control that distinct genes exhibit at the level of initiation, suggesting that factors influencing 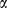, such as secondary structure in the 5′ leader region, have been shaped by evolution to contribute to the delicate balance of cellular protein composition.

To show that the procedure introduced above can be applied under different conditions, we carry out a similar analysis under pheromone treatment by using the corresponding measurements of ribosome densities from [23], and estimate the initiation rates 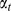 under these conditions. The initiation rates do not substantially change, consistent with the finding by Mackay et al. [23] that only a small number of mRNAs exhibit altered densities after pheromone treatment. However, with our analysis we identify two mRNAs, *SAG1* and *HO*, which exhibit a radical change in their initiation rate value under pheromone treatment. Importantly, these two mRNAs have been shown to present altered 5′UTR sequences that explain their significant ribosome density change [24].

### Translation initiation rates correlate with the lengths of the transcripts

Now we analyse the influence of the physical properties of the mRNA on the translation initiation rate by analysing the correlations of the identified 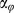 rates with the presence of secondary structures in the 5′UTR and the length of the transcript. The physiological estimates of the initiation rate show a small but significant correlation with the free energy of the secondary structures in the 5′UTR (Spearman's rank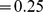, p-value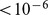) confirming that secondary structures may have an important regulatory role, as already suggested [9], [25], [26], see Supplementary Information Text S1.

**Figure 3:**
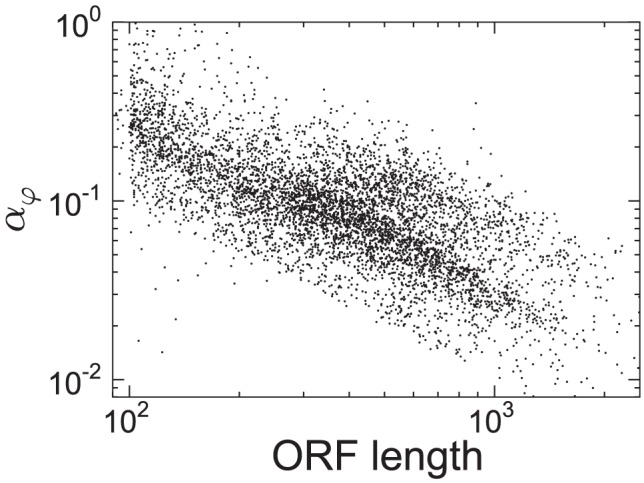
Scatter plot of the ORF lengths 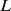 against the estimated 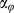
's. The log-log scatter plot shows possible signatures of a power-law dependence.

Remarkably, we find a strong negative correlation between the initiation rate and the length of the ORF (Spearman's rank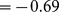, p-value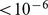), see Figure 3. Of relevance to this observation, Arava and coworkers [7] found that ribosome density counter-intuitively and systematically decreases with increasing the ORF length. In a subsequent work [27], they reported that the explanation most consistent with their experimental investigation was that lower initiation rates predominate on longer mRNAs, exactly as we estimate in this work.

### mRNA-specific responses of ribosome traffic correlate with gene ontology

#### *Smooth* and *abrupt* sequences

**Figure 4:**
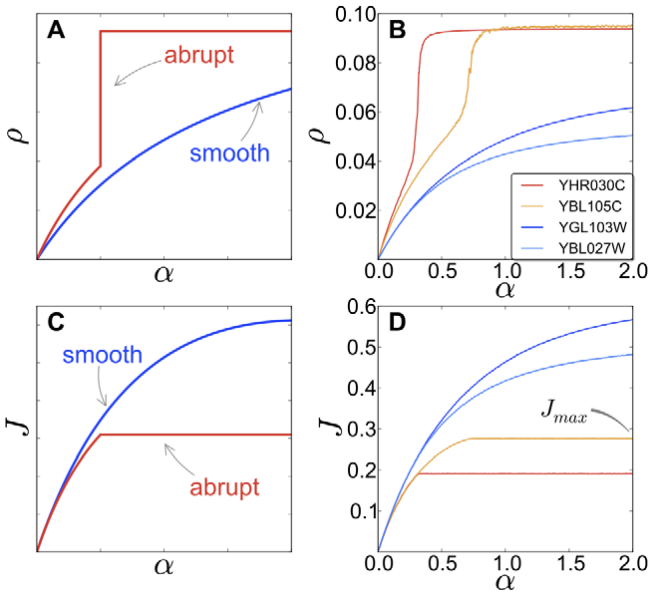
Outcomes for some mRNAs obtained by stochastic simulations of the model. Panels (**A**) and (**C**) show a sketch of the two different behaviours one can obtain for the density of ribosomes 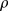 and the current 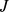, respectively. The genes are divided in two categories, according to the shape of 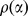, as shown in (**A**): *abrupt* mRNAs (red, colour online) present a steep increase of the polysome size with increasing the initiation rate. On the other and, *smooth* sequences (blue, colour online) do not show this feature. The current (**C**) is also affected, with *abrupt* genes exhibiting a sudden change, or ‘kink’ in the current, while the current of *smooth* mRNAs does not suddenly saturate. Panels (**B**) and (**D**) show the outcome of numerical simulations of real sequences from *S. cerevisiae*. Genes YGL103W and YBL027W are ribosomal proteins while YHR030C and YBL105C are kinase regulatory proteins. 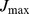 indicates the saturation value of the current (see text).

Our genome-wide simulation generates, for each transcript, curves describing how the ribosome density 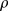 (polysome size) and the ribosomal current 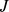 depend on the initiation rate 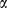. According to these characteristic curves, mRNA sequences fall predominantly into either of two categories (Figure 4): the ribosome density of some mRNAs presents a steep increment with increasing 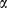 (we refer to these sequences as *abrupt*), while others present a gradual increase of the density with the initiation rate (*smooth* sequences). This classification coincides with the characteristic curve obtained for the ribosomal current 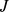 against 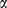. In *abrupt* sequences the current presents a kink before reaching its saturation value, whereas in *smooth* sequences, the saturation value of 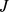 is reached gradually [28]. Therefore, mRNA sequences can be classified into two different types depending on how their overall translation rate and polysome size vary upon changes in the initiation rate.

The origin of these two types of ribosome traffic lies in the codon arrangement: *smooth* behaviour mRNAs contain either rare codons at the 5′ end, or no rare codons at all, whereas *abrupt* behaviour mRNAs contain rare codons or clusters of rare codons within the main body of the mRNA. These rare codons act as bottlenecks, causing queues to build up and consequently 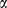-dependent abrupt phase transitions to occur. In contrast, if the bottleneck is right at the beginning of the mRNA, no queue can be formed and therefore the polysome size increases smoothly with the initiation rate 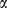 [28].

The curves for the ribosome density versus 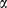 for the more than 6,000 *S. cerevisiae* mRNAs were analysed with an automated clustering algorithm [29] to classify them into *abrupt* or *smooth* sequences. The algorithm clearly classified 35% sequences to belong to the *abrupt* category, and 38% to the *smooth* category. The remaining 27% sequences were marked as *hybrid* since they did not show pronounced features to justify a discrimination between the two categories (Supplementary Information Text S1).

Strikingly, these two categories, each with distinct initiation rate response criteria (smooth or abrupt) correlate with the biological function of the encoded proteins: GO annotations (process) related to translation are significantly over-represented in *smooth* sequences (*cytoplasmic translation*, P-value 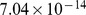, *translation*, P-value 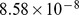). Conversely, *abrupt* sequences are connected to several processes, mainly involving regulation, e.g. *biological regulation*, P-value 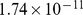, *metabolic process*, P-value 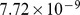 or *cellular response to stimulus*, P-value 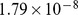. More details about the enrichment in each category can be found in the Supplementary Table S3.

If one considers the abundance of the transcripts in the cell (data from [30]), then 68% of the total mRNA population belongs to the *smooth* type. This indicates that highly transcribed genes have preferentially slow codons at the 5′ end rather than in the main body of the mRNA. In this way, highly transcribed genes avoid having queues of ribosomes which might deplete a large amount of essential cell resources. *Abrupt* sequences, on the other hand, constitute only 14% of the transcribed mRNAs; this is consistent with the fact that *abrupt* sequences typically encode regulatory proteins, which are in general of low abundance.

#### Responsiveness of translation rate to changes in initiation rate

A further characteristic we can conjecture from our genome-wide simulations, is how the translation rate changes upon variations in the initiation rate around the physiological value 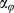 quantified above. As previously mentioned, the value of the translation initiation rate 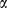 depends on several factors, such as the amount of free ribosomes, initiation factors, and folding features of the 5′ UTRs, all of which are strongly influenced by stress and nutrient conditions [31]. The knowledge of the responsiveness of translation rate to variations of the initiation rate, therefore, theoretically provides key insight into the mechanisms of translational regulation of gene expression.

**Figure 5:**
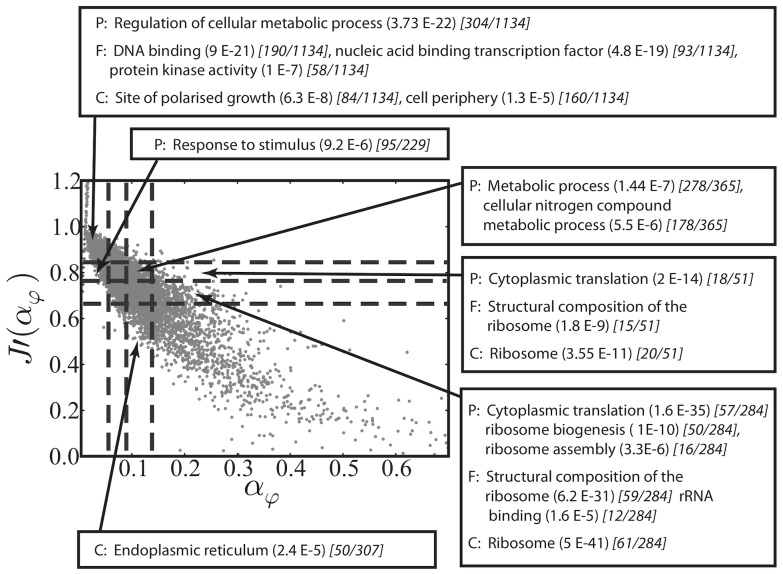
Scatter plot of the estimated initiation rates 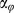 versus the slope of the protein production rate 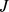 evaluated at 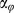. Both distributions of initiation rates and slopes have been subdivided in quartiles (dashed lines), defining 16 regions. Boxed annotations indicate those GO categories that are overrepresented in each quartile sector (P; GO process, F; function, C; component) with the P-value indicated as a power 10 exponent (E). The enrichments in each region are indicated in the square brackets as xx/yy, where ‘xx’ is the number of genes with that specific annotation and ‘yy’ the total number of genes in the region.

In order to study translation rate change responsiveness, or ‘gearing’, we study the combined role of 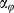 and the presumed gradient 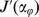 of the translation rate, which quantifies the responsiveness of 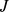 around the physiological value of the initiation rate (see ‘Materials and Methods’). We find that these two quantities are highly correlated (Spearman's rank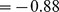, p-value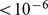), Figure 5. This indicates that, according to our model, genes characterised by a small initiation rate, such as regulatory genes, have in general a high translation rate gradient, suggesting that the corresponding proteins are produced at low levels under normal conditions but their synthesis can be rapidly increased upon changes in the initiation rate. Conversely, genes characterised by a high initiation rate, such as genes encoding proteins involved in translation and ribosome biogenesis, exhibit in general a medium-to-low value of the translation rate responsiveness, implying that their synthesis is tuned to be efficient, but stable against variations in initiating ribosomal subunit availability. Moreover, by dividing the distributions into quartiles, we identify sixteen different regions; a number of them exhibit significant enrichment in specific GO annotations determined principally on the basis of 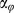 (Figure 5). However, by constraining the genes analysed to those with an 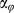 value lying within a specific range, the specific contribution of 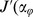) could be identified. This revealed that there is a further, separable enrichment of GO categories on the basis of 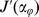. This in turn indicates that the gearing function, or responsiveness to ribosome availability, is also coupled to gene function. Thus regions 4, 7, 8, 12 and 16 from Figure 5 (regions are numbered starting from the top left one and proceeding left to right) show a significant enrichment in specific GO annotations with a P-value smaller than 0.01 (see Supplementary Table S4). The genes exhibiting a significant enrichment in region 16 were un-annotated. The results in Figure 5 (the estimated 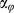 plotted against the 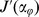 values) are annotated with GO category enrichments influenced by the combination of the physiological initiation rate and the gearing factor.

We would like to emphasise that, unlike all other results shown in this work, the gearing factor 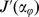, i.e. the responsiveness capacity of the mRNAs, remains a speculative and theoretical outcome of the model. Since genome-wide experimental setups changing the initiation rates of single transcripts and observing the variation of translation remain are nowadays a challenge, its biological relevance remains to be proven.

#### Maximal translation rate

We also perform a genome-wide analysis of the maximal translation rate 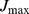 that a sequence can achieve, when an increase in the initiation rate 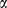 does not yield any further change in 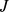 (see ‘Materials and Methods’). We extract 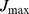 for each mRNA sequence from our genome-wide simulations and analyse the mRNAs with largest and smallest 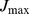 (first quartiles). We find that sequences with the largest maximal production rate are mRNAs involved in cytoplasmic translation (P-value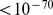), such as ribosomal and translational proteins. Conversely, proteins with regulatory functions (such as *nucleic acid binding transcription factor activity, P-value* 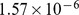) are encoded by sequences with the smallest 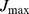. Supplementary Table S5 summarises this GO analysis.

In summary, our genome-wide analysis shows that the type of ribosome traffic on mRNA is significantly correlated with the biological function of the encoded protein: essential proteins that need to be constitutively produced, such as ribosomal proteins and proteins involved in translation, typically exhibit a smooth increase of polysome size upon increments of the initiation rate, a large physiological initiation rate and a high maximal overall translation rate. In contrast, proteins such as the ones involved in responses to stimuli typically exhibit an abrupt increase of the polysome size with the initiation rate, present a small physiological initiation rate and a low maximal overall translation efficiency. In the next section we discuss the fundamental role of codon arrangement in determining the translation efficiency.

### Codon arrangement *versus* codon usage

**Figure 6:**
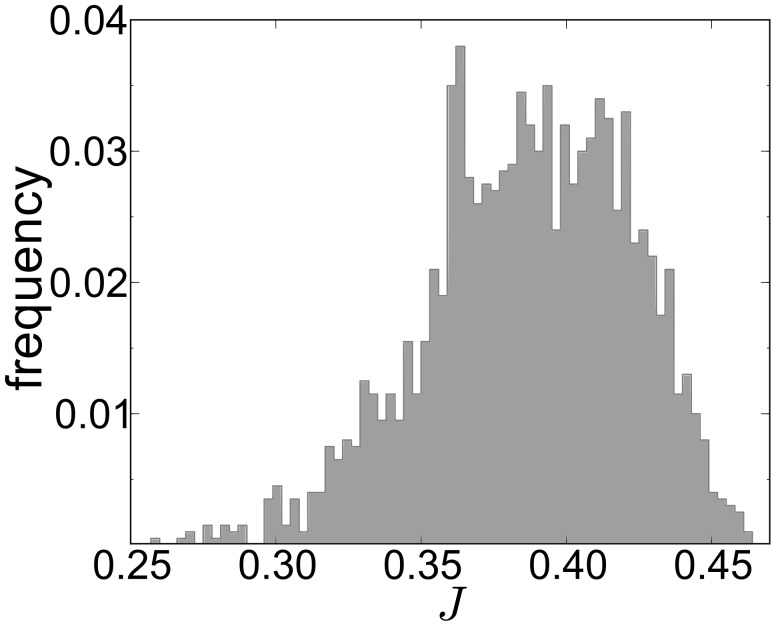
Normalised histogram of the simulated protein production rates of the YPL106C randomised ensemble. We constructed the randomised ensemble by shuffling the YPL106C codon choice at each sequence position, generating 2,000 different variants, each time keeping the amino acid sequence and overall codon composition constant. For example, for the chosen gene, CAI = 0.521. The value of the chosen initiation is 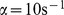.

In order to show that the genome-wide correlation between translational efficiency and biological function obtained above is not only the consequence of codon usage but is strongly influenced by the order in which codons are used in the mRNA, we simulate the translation of a library of randomised ORFs such that both amino acid sequence and codon composition remain identical. That means, two ORFs belonging to the library have exactly the same codon usage but the arrangement of these codons is different. Here we show that, even though all these randomised ORFs have exactly the same codon usage indices such as the CAI, codon adaptation index [32], and tAI, tRNA adaptation index [33], their predicted protein production rate can be very different. Figure 6 shows how the different values of predicted protein production rate (ribosomal current 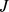 for a fixed initiation rate) are distributed for 2,000 synonymous randomised codon sequences of a typical *Saccharomyces cerevisiae* gene (YPL106C).

Figure 6 clearly shows that the relative positioning of codons has a crucial effect on the translation efficiency, suggesting that very different cellular production rates can be achieved through evolution of the codon arrangement. For instance, in the case shown in Figure 6 there is an increase of about 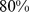 from the lowest to the highest value of the translation rate. The variation of 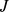 for different codon arrangements is a general result and does not depend on the gene or the chosen initiation rate 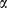 (for more information see Supplementary Information, Text S1). We thus show that by randomising codon arrangement (i.e. randomly exchanging the position of synonymous codons in a sequence), different protein production rates are obtained, even though codon usage remains fixed. This indicates that the codon arrangement has a highly significant role in determining the efficiency of translation.

### Validation of genome-wide translation rate prediction by experimental data

While several models of protein synthesis have been developed over the last decades [34], the role of codon sequence and stochastic ribosomal movement has been investigated only recently. But even recent models typically treat the initiation rate as a fixed parameter, identical for all mRNAs, despite its key role in determining translational efficiency. In contrast, our model predicts the protein production rate 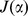 as a function of the initiation rate. By then integrating genome-wide simulations with datasets of polysome sizes, we have identified the physiological value of the initiation rate 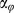 for each mRNA. This set of values 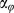 then leads to the prediction of the protein production rate 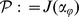 for each transcript. This allows us to validate our model predictions with experimental data.

**Figure 7:**
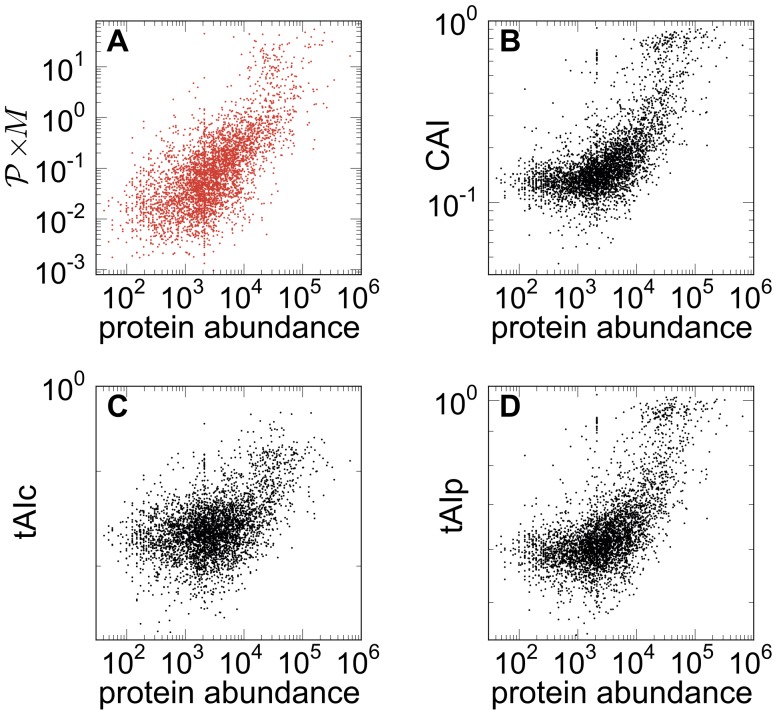
Scatter plots of different estimators of protein production rates. (**A**) 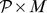 versus abundance of proteins. The mRNA abundances are from [30] and the experimentally measured protein levels from [51]. The plot shows a clear correlation between the model prediction of the amount of proteins in the cell and the experimental values. (**B**) CAI from [30] versus protein abundance. (**C**) and (**D**) show different variants of the tRNA adaptation index, tAIc and tAIp from [30], vs protein abundance. Our approach yields a better correlation between the predicted and measured protein abundance.

Figure 7A is a scatter plot of the genome-wide simulations versus measured protein abundance from [30]. The model predictions for 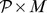, where 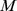 denotes mRNA abundance, correlate very well with the experimental protein abundances (Spearman's rank = 0.64, p-value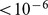), compared to other attempts such as the tAIc (Spearman's rank = 0.38, p-value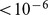). Our outcome is further improved when considering just transcripts loaded onto polysomes (Spearman's rank = 0.66, p-value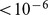), see ‘Materials and Methods’ and Supplementary Information, Table S1. Moreover, as it can be appreciated from Figure 7A, the predictions from our model correlate very well with measured protein abundance for all ranges of gene expression, in contrast to other translation efficiency indices (panels B,C,D), which exhibit a poor correlation for lowly expressed genes.

## Discussion

The phenotype exhibited by any cell is dictated by its proteomic composition. How much of each type of protein is expressed is governed by a range of factors, including the level of transcription and stability of the encoding mRNA, the half-life of the protein, and how efficiently its mRNA is translated. A number of strategies have been employed to predict translational efficiency, many of which utilise the observation that not all codons are used with equal frequency, and that codon usage frequency is proportional, at some level, to the abundance of the corresponding decoding tRNA species [35], [36]. Initially, measures such as the codon adaptation (CAI) index were developed [32], which correlate high protein abundance with over-use of the sub-set of codons found in a group of very-highly expressed genes, normally those encoding the ribosomal proteins. However, such approaches frequently struggle to predict the expression level of less abundant proteins. More recently, dynamic TASEP (Totally Asymmetric Simple Exclusion Process) models have been employed to simulate the flow of ribosomal traffic, including queuing interactions between adjacent ribosomes on the polysome [20], [37]–[41]. Even though these models represent a big step towards a more complete description of the translation process, most of them miss one essential component, namely the mechano-chemical ribosome cycle. By including this mechanism into an exclusion process we showed that the mathematical description of translation becomes much more accurate [16]. Here we applied this model to simulate the translation of every mRNA in the transcriptome of *S. cerevisiae* leading to the estimates of the individual translation initiation rates unique to each of the 6,000 genes in yeast. We furthermore showed that mRNA sequences can be classified according to their ribosome traffic characteristics, and crucially, this classification maps to gene ontology assignments.

Even though the role of the translation initiation rate has been shown to play a central role in translational control of gene expression [9], to our knowledge no genome-wide estimations of these rates have been reported, considering ribosome traffic effects. The translation initiation rate, i.e. the rate at which ribosomes start translating the ORF, condenses many factors, such as cytoplasmic ribosome availability, initiation factors and secondary structures on the 5′UTRs, all of them strongly dependent on nutrients and stress conditions. Some approaches consider the translation initiation rate to be fixed for every transcript, thereby neglecting the key factors that make the initiation rate unique to each transcript. In contrast, by considering traffic dynamics, we determined the first genome-wide estimate of initiation rates 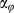 for each and every mRNA (Figure 2) by integrating our stochastic model of ribosome traffic with data of ribosome densities across all mRNAs [23]. Our analysis showed a wide range of 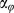 values under these non-stress conditions. Importantly, the 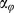 values are strongly correlated with gene function, explaining for example why translation of ribosomal protein mRNAs, which typically have a very high 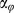 value, is very efficient. These values of 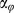 are expected to be influenced by the degree of secondary structure of the 5′ leader sequence, and indeed we did find a significant correlation with the free energies of the secondary structures. The strongest connection involving 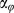 was however a negative correlation with mRNA length, mirroring the findings from experimental research that described lower ribosome densities on longer mRNAs [7], [15]. In contrast to the explanation that the effect could be caused by bottlenecks of slow codons [15] (see Supplementary Information Text S1) this negative correlation supports the idea that due to the circular structure of mRNAs, the ends of shorter mRNAs can interact more easily than longer mRNAs, thereby promoting ribosome recycling [7], [42], [43]. Indeed following detailed experimental analysis using ribosome density mapping, Arava and colleagues concluded that lower densities on longer mRNAs are best explained by lower rates of translation initiation [27], mirroring our findings in this work. To summarise, we interpret the correlation between the estimated initiation rates and the ORF lengths as a possible indication of a regulatory mechanism that allows circularised mRNAs to load ribosomes more efficiently onto their transcripts, leading to the observed ribosome-ORF length relationship.

Our analysis furthermore identified two main distinct classes of mRNAs regarding their responsiveness to changes in the initiation rate 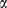: some sequences exhibited an *abrupt* change in the polysome size upon a change in 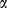, whereas *smooth* sequences showed a gradual increase. Calculations with artificial sequences revealed that sequences with rare codons in the main body of the ORF belong to the *abrupt* class, whereas sequences with either no rare codons or rare codons at the 5′ end belong to the *smooth* class [28]. Crucially, we note that the classification of mRNAs into *smooth* and *abrupt* responders maps onto particular gene ontological classifications. *Smooth* responder mRNAs as a class are highly over-populated with ribosomal protein mRNAs and translation factors. Conversely, the *abrupt* class contains disproportionate numbers of regulatory proteins, including nucleic acid-binding transcription factors, and cell cycle proteins. One reason why ribosomal protein mRNAs are predominantly of the *smooth* response type might relate to the massive manufacturing scale of ribosome biosynthesis; in yeast, ribosomal protein mRNAs account for nearly 30 percent of all mRNAs [44], [45]. *Smooth*-type responses to 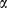 must be of selective advantage for a cell, since if ribosome queues were established on such a large proportion of the cell mRNA population they would sequester a large numbers of ribosomes, with deleterious consequences for cell fitness. On the other hand, it has been recently found that cell-cycle regulated genes predominantly adopt non-optimal codon usage (with no ramp of slow codons at the beginning, and therefore of the *abrupt* class) to achieve elongation-limited mRNA translation; this can generate cell cycle-dependent oscillations in protein abundance induced by changes in the tRNA pool [46]. Therefore, it is apparent that the cell coordinates codon usage and codon arrangement to achieve translational gene expression control.

**Figure 8:**
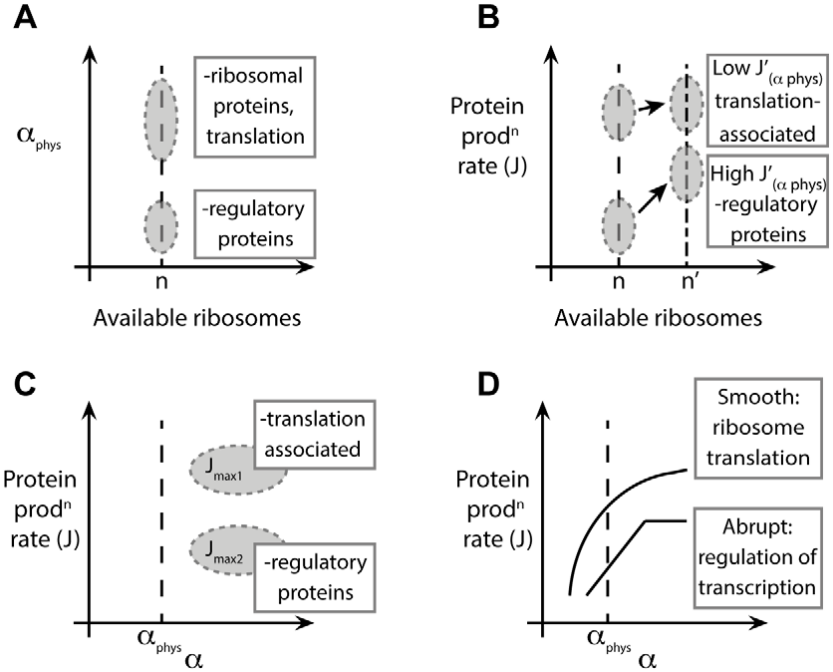
Initiation rate: summary of the findings. (**A**) For a given ‘physiological’ number of ribosomes 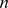 we found mRNA-specific initiation rates, distributed over a broad range of values (Figure 2). Different regions of the distributions can be mapped to certain GO annotations. For example, mRNAs with small physiological initiation rate 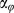 are regulatory proteins while genes involved in translation have a larger initiation rate. (**B**) Changes in initiation (induced, for instance, by variations in the ribosomal pool, e.g. available ribosomes increase to a value of 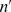) are estimated by our modelling and theoretically perceived by the transcript in different ways, according to their current-initiation relationship 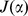. In particular, some mRNAs have a large gearing factor 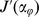, such as regulatory proteins, while other messengers, such as translation associated ones, are less sensitive to changes of the initiation rate. (**C**) For very large initiation rates the protein production rates reach a maximal elongation-limited value, i.e. only depending on the sequence of codons. We discover that translation associated genes have a larger maximal production rate when compared to other mRNAs, such as regulatory proteins, whose production might need to be capped. (**D**) In general we find two main groups of sequences classified according to their current-initiation relationship 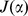. Abrupt sequences, usually regulatory proteins, present an abrupt ‘kink’ in 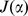, meaning that the protein production rate can quickly saturate above specific values (sequence-dependent) of the initiation rate. Genes involved in translation like ribosomal proteins are instead classified as smooth sequences, since their sequences are such that this abrupt crossover does not exist.

Our results also showed important differences in the computationally deduced slope of the 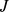 production rate curve in response to increasing 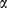. Some mRNAs are what we term highly geared, that is, small increases in 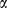 produce relatively large increases in 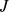. This type of super-responsive mRNA was significantly enriched in regulatory proteins, which also have a relatively small initiation rate. We speculate that this might be a mechanism to facilitate rapid responses to changed environmental conditions, allowing, for example, rapid synthesis of transcriptional repressors that in physiological conditions are severely limited by the initiation (low 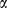). Conversely, low geared mRNAs, where increases in 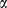 produce proportionately lower responses in 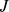, were enriched in ribosomal proteins. Since ribosomal proteins are used to manufacture ribosomes, lower gearing of the 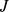 responsiveness to 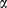 may help prevent undesirable positive feedback effects. We furthermore classified mRNA sequences according to the maximal translation rate that they can achieve, i.e. their saturation value, and our analysis revealed that *abrupt* sequences have predominantly a small 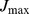, whereas *smooth* sequences are characterised by a large 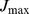. This correlates with the levels of the corresponding proteins: regulatory proteins are typically present in low abundance, whereas ribosomal proteins are highly abundant. Moreover, this might prevent possibly deleterious consequences of over-producing regulatory proteins, including cell cycle factors, during occasional bursts of ribosomal availability that would lead to a very large increase in the value of 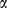. In *S. cerevisiae* for example, this occurs upon sudden glucose depletion: translation initiation is rapidly inhibited [47] but some mRNAs (including those involved in carbohydrate metabolism) continue to be translated [48], thereby being exposed to a spike in ribosome availability. Similar complex translational re-programming, coincident with a partial cell-wide shut down of translation initiation, occurs in response to oxidative stress [49]. Hence, by having a high responsiveness to 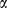 and a low 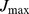, *abrupt* sequences can have a very rapid gene expression upregulation, on one hand, but a controlled maximum translation rate, on the other hand. Figure 8 summarises our findings on the initiation rates and the consequences of different (mRNA-specific) dependencies of the protein production rate on the initiation step.

Incidentally, this classification of proteins according to their translation dynamics, coincides with the classification according to protein stability. In [50], the *S. cerevisiae* proteome was analysed using a clustering approach to classify proteins according to half-life, and the stable protein cluster was enriched with proteins involved in protein production, including ribosomal proteins and enzymes involved in amino acid metabolism. Moreover, the unstable protein cluster was enriched with cell cycle proteins and proteins involved in transcriptional regulation. Therefore, our analysis indicates that stable proteins tend to have a low responsiveness in their production rate to external changes which change the initiation rate, whereas unstable proteins production responses very effectively to external changes. Hence, our analysis strongly suggests that the cell coordinates dynamics of protein degradation with the dynamics of protein production.

In summary, we have shown how our stochastic model representing the ribosome traffic flow on mRNAs is able to discern and describe the biological interplay between translation initiation and elongation, at a single-codon level. We have illustrated how the application of this model across the entire genome can be used to infer mRNA-specific translation initiation rates *in vivo*, and that selection of codon arrangement is likely to be an important mechanism to tune the translation system to meet the competing demands of ribosome biosynthesis and translation of all other mRNAs in the cell. With our approach, mRNA sequences can be classified according to their translation dynamics, mapping to key gene ontological classifications; codon arrangement plays a fundamental role in this classification, indicating that it is optimized through evolution to match the corresponding gene function. Moreover, gene-specific physiological values of initiation rate can be used to determine the translational efficiency for each mRNA; this allows the prediction of genome-wide protein abundances with a significant increase in correlation when compared with previous approaches (Figure 7). We foresee this type of analysis will be of great value to understand how the economics of translation are regulated on a cell-wide basis, and how codon arrangement is optimised to control gene expression in response to the translational remodelling that occurs in response to many environmental stresses.

## Materials and Methods

### Stochastic simulations

For each mRNA sequence of *S. cerevisiae* we performed a stochastic simulation of translation, one mRNA at a time, following the rules explained above and summarised in Figure 1. Our algorithm is a continuous time Monte-Carlo based on the Gillespie algorithm, and therefore it gives the real-time dynamics of the system.

In each simulation of individual mRNAs we let the system reach the steady-state. Then we measured, at constant interval times, two quantities: the current 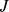 of ribosomes along the mRNA, i.e., how many ribosomes per unit time finish translation, and the density 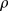 of ribosomes on the mRNA, i.e., the total number of ribosomes 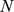 divided by the length 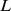 (in codons) of the mRNA. Therefore, the current 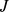 gives the translation rate, and the density 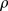 determines the polysome size. We then averaged these quantities over the entire time interval of the simulation. We ran the simulations for a broad range of initiation rates 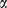 between 0 and 5 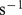, making sure that the plausible physiological regime for 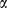 variation was covered, and we fixed the other parameters as explained in the previous sections. The obtained curves 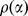 and 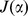 were then smoothed with a ten-points running average.

The gradient 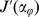 of the translation rate at the physiological initiation rate is defined, for each mRNA, as the numerical derivative of the relation 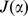, computed at 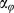. It geometrically represents the slope of the curve 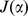 at the physiological value 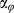. Since both the distributed physiological values of the initiation rates and different codon sequences cause a different dependence of 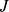 on 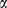, the derivative 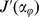 differs from mRNA to mRNA.

The maximal values 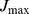 and 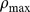 were defined as the mean of the last five simulation points of the current and the density, respectively, corresponding to the five largest values of 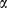 considered.

### Translation rate prediction

The translation efficiency 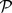 of a transcript is defined as the protein production rate 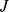 computed at the physiological value 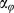, 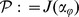. Denoting by 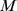 the amount of a specific mRNA in the cell (data from [30]), for any protein the quantity 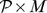 is an estimate of the protein abundance, see [20]. We also considered the effective amount of transcript involved in polysomes, 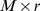, where 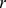 can be found in [23]. The prediction 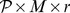 slightly improves the correlation with measured protein abundance, as discussed in the ‘Results’ section.

## Supporting Information

Table S1 Dataset for the classification of the transcripts in the abrupt, smooth and hybrid classes, and values of the physiological and maximal quantities (initiation rates and ribosomal current) characteristic of each mRNA. In the second sheet one can find the database for the different estimators of protein production rates used in Fig. 7.
(XLS)

Table S2 GO annotations of genes found in regions (i)–(iv) of Figure 2. Each sheet is named with the corresponding region (i)–(iv) of Figure 3, and with the GO aspect (process, function, component).
(XLS)

Table S3 GO annotations of genes found in the smooth, abrupt and hybrid classes. Each sheet is named with the sequence type (smooth, abrupt, hybrid), and with the GO aspect (process, function, component).
(XLS)

Table S4 GO annotations of genes found in different regions of Figure 5. Each sheet is named with the corresponding region (4-7-8-12-16) of Figure 5, and with the GO aspect (process, function, component).
(XLS)

Table S5GO annotations of genes belonging to the top and bottom quartile of the 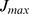 distribution. Each sheet is named according to their 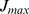 (top quartile = 25% of genes having the largest 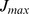, bottom quartile = 25% of genes having the smallest 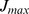), and with the GO aspect (process, function, component).(XLS)

Text S1 Detailed description of the approaches used in the main text and regarding (1) the estimate of the hopping rates in the two-state model; (2) the method to classify sequences in abrupt, smooth and hybrid class; (3) other examples of randomisation (with constant amino-acid sequence) of codon arrangement and expected protein production rates (similar to Figure 6); (4) numerical details for the quantification of the initiation rates; (5) details on the computed energy of secondary structures ; (6) an accurate explanation of the alternative hypothesis for the ORF-length dependence of the initiation rate; (7) description of the computation of the p-values.
(PDF)

Text S2 Comparison with the previous model by Siwiak and Zielenkiewicz [22].
(PDF)

## References

[1] Spellman PT, Sherlock G, Zhang MQ, Iyer VR, Anders K, et al (1998) Comprehensive identification of cell cycle-regulated genes of the yeast saccharomyces cerevisiae by microarray hybridization. Molecular Biology of the Cell 9: 3273–3297. 9843569 10.1091/mbc.9.12.3273 PMC25624

[2] Shalon D, Smith SJ, Brown PO (1996) A DNA microarray system for analyzing complex DNA samples using two-color uorescent probe hybridization. Genome Research 6: 639–645. 8796352 10.1101/gr.6.7.639

[3] Ideker T, Thorsson V, Ranish JA, Christmas R, Buhler J, et al (2001) Integrated genomic and proteomic analyses of a systematically perturbed metabolic network. Science 292: 929–934. 11340206 10.1126/science.292.5518.929

[4] Schwanhausser B, Busse D, Li N, Dittmar G, Schuchhardt J, et al (2011) Global quantification of mammalian gene expression control. Nature 473: 337–342. 21593866 10.1038/nature10098

[5] Ghazalpour A, Bennett B, Petyuk VA, Orozco L, Hagopian R, et al (2011) Comparative analysis of proteome and transcriptome variation in mouse. PLoS Genet 7: e1001393. 21695224 10.1371/journal.pgen.1001393 PMC3111477

[6] Ghaemmaghami S, Huh W, Bower K, Howson RW, Belle A, et al (2003) Global analysis of protein expression in yeast. Nature 425: 737–741. 14562106 10.1038/nature02046

[7] Arava Y, Wang Y, Storey JD, Liu CL, Brown PO, et al (2003) Genome-wide analysis of mRNA translation profiles in saccharomyces cerevisiae. Proc Natl Acad Sci U S A 100: 3889–3894. 12660367 10.1073/pnas.0635171100 PMC153018

[8] Gingold H, Pilpel Y (2011) Determinants of translation efficiency and accuracy. Mol Syst Biol 7: 481. 21487400 10.1038/msb.2011.14 PMC3101949

[9] Kudla G, Murray AW, Tollervey D, Plotkin JB (2009) Coding-sequence determinants of gene expression in escherichia coli. Science 324: 255–258. 19359587 10.1126/science.1170160 PMC3902468

[10] Livingstone M, Atas E, Meller A, Sonenberg N (2010) Mechanisms governing the control of mRNA translation. Physical Biology 7: 021001. 20463379 10.1088/1478-3975/7/2/021001

[11] Kozak M (1989) Circumstances and mechanisms of inhibition of translation by secondary structure in eucaryotic mRNAs. Mol Cell Biol 9: 5134–5142. 2601712 10.1128/mcb.9.11.5134-5142.1989 PMC363665

[12] Sagliocco FA, Vega Laso MR, Zhu D, Tuite MF, McCarthy JE, et al (1993) The inuence of 5′-secondary structures upon ribosome binding to mRNA during translation in yeast. J Biol Chem 268: 26522–26530. 8253781

[13] Buchan JR, Stansfield I (2007) Halting a cellular production line: responses to ribosomal pausing during translation. Biology of the Cell 99: 475–487. 17696878 10.1042/BC20070037

[14] Tuller T, Carmi A, Vestsigian K, Navon S, Dorfan Y, et al (2010) An evolutionarily conserved mechanism for controlling the efficiency of protein translation. Cell 141: 344–354. 20403328 10.1016/j.cell.2010.03.031

[15] Ingolia NT, Ghaemmaghami S, Newman JRS, Weissman JS (2009) Genome-wide analysis in vivo of translation with nucleotide resolution using ribosome profiling. Science 324: 218–23. 19213877 10.1126/science.1168978 PMC2746483

[16] Ciandrini L, Stansfield I, Romano MC (2010) Role of the particle's stepping cycle in an asymmetric exclusion process: A model of mRNA translation. Physical Review E 81: 051904. 10.1103/PhysRevE.81.051904 PMC3639468 20866258

[17] Percudani R, Pavesi A, Ottonello S (1997) Transfer RNA gene redundancy and translational selection in saccharomyces cerevisiae. Journal of Molecular Biology 268: 322–330. 9159473 10.1006/jmbi.1997.0942

[18] Savelsbergh A, Katunin VI, Mohr D, Peske F, Rodnina MV, et al (2003) An elongation factor g-induced ribosome rearrangement precedes tRNA-mRNA translocation. Mol Cell 11: 1517–23. 12820965 10.1016/s1097-2765(03)00230-2

[19] Chou T, Mallick K, Zia RKP (2011) Non-equilibrium statistical mechanics: from a paradigmatic model to biological transport. Reports on Progress in Physics 74: 116601.

[20] Reuveni S, Meilijson I, Kupiec M, Ruppin E, Tuller T (2011) Genome-Scale analysis of translation elongation with a ribosome ow model. PLoS Comput Biol 7: e1002127. 21909250 10.1371/journal.pcbi.1002127 PMC3164701

[21] Wen J, Lancaster L, Hodges C, Zeri A, Yoshimura SH, et al (2008) Following translation by single ribosomes one codon at a time. Nature 452: 598–603. 18327250 10.1038/nature06716 PMC2556548

[22] Siwiak M, Zielenkiewicz P (2010) A Comprehensive, Quantitative, and Genome-Wide Model of Translation. PLoS Comput Biol 6 7: e1000865. 20686685 10.1371/journal.pcbi.1000865 PMC2912337

[23] MacKay VL, Li X, Flory MR, Turcott E, Law GL, et al (2004) Gene expression analyzed by high-resolution state array analysis and quantitative proteomics. Molecular & Cellular Proteomics 3: 478–489. 14766929 10.1074/mcp.M300129-MCP200

[24] Law GL, Bickel KS, MacKay VL, Morris DR (2005) The undertranslated transcriptome reveals widespread translational silencing by alternative 5′ transcript leaders. Genome Biology 6: R111. 16420678 10.1186/gb-2005-6-13-r111 PMC1414110

[25] Ringnér M, Krogh M (2005) Folding free energies of 5′-UTRs impact Post-Transcriptional regulation on a genomic scale in yeast. PLoS Computational Biology 1: e72. 16355254 10.1371/journal.pcbi.0010072 PMC1309706

[26] Tuller T, Waldman YY, Kupiec M, Ruppin E (2010) Translation efficiency is determined by both codon bias and folding energy. Proceedings of the National Academy of Sciences 107: 3645–3650. 10.1073/pnas.0909910107 PMC2840511 20133581

[27] Arava Y, Boas FE, Brown PO, Herschlag D (2005) Dissecting eukaryotic translation and its control by ribosome density mapping. Nucleic Acids Research 33: 2421–2432. 15860778 10.1093/nar/gki331 PMC1087779

[28] Romano MC, Thiel M, Stansfield I, Grebogi C (2009) Queueing phase transition: Theory of translation. Physical Review Letters 102: 198104. 19519001 10.1103/PhysRevLett.102.198104 PMC3639427

[29] Press W, Teukolsky S, Vetterling W, Flannery B (2007) Numerical Recipes 3rd Edition: The Art of Scientific Computing. Cambridge Univ Press.

[30] Beyer A, Hollunder J, Nasheuer H, Wilhelm T (2004) Post-transcriptional expression regulation in the yeast saccharomyces cerevisiae on a genomic scale. Molecular & Cellular Proteomics 3: 1083–1092. 15326222 10.1074/mcp.M400099-MCP200

[31] Mayer C, Grummt I (2006) Ribosome biogenesis and cell growth: mTOR coordinates transcription by all three classes of nuclear RNA polymerases. Oncogene 25: 6384–6391. 17041624 10.1038/sj.onc.1209883

[32] Sharp PM, Li WH (1987) The codon adaptation index-a measure of directional synonymous codon usage bias, and its potential applications. Nucleic Acids Res 15: 1281–1295. 3547335 10.1093/nar/15.3.1281 PMC340524

[33] Reis Md, Savva R, Wernisch L (2004) Solving the riddle of codon usage preferences: a test for translational selection. Nucleic Acids Research 32: 5036–5044. 15448185 10.1093/nar/gkh834 PMC521650

[34] von der Haar T (2012) Mathematical and computational modelling of ribosomal movement and protein synthesis: an overview. Computational and Structural Biotechnology Journal 1. 10.5936/csbj.201204002 PMC3962216 24688632

[35] Ikemura T (1982) Correlation between the abundance of yeast transfer RNAs and the occurrence of the respective codons in protein genes: Differences in synonymous codon choice patterns of yeast and escherichia coli with reference to the abundance of isoaccepting transfer RNAs. Journal of Molecular Biology 158: 573–597. 6750137 10.1016/0022-2836(82)90250-9

[36] Dong H, Nilsson L, Kurland CG (1996) Co-variation of tRNA abundance and codon usage in escherichia coli at different growth rates. Journal of Molecular Biology 260: 649–663. 8709146 10.1006/jmbi.1996.0428

[37] Sharma AK, Chowdhury D (2011) Stochastic theory of protein synthesis and polysome: Ribosome profile on a single mRNA transcript. Journal of Theoretical Biology 289: 36–46. 21888920 10.1016/j.jtbi.2011.08.023

[38] Mitarai N, Sneppen K, Pedersen S (2008) Ribosome collisions and translation efficiency: optimization by codon usage and mRNA destabilization. J Mol Biol 382: 236–245. 18619977 10.1016/j.jmb.2008.06.068

[39] Garai A, Chowdhury D, Chowdhury D, Ramakrishnan TV (2009) Stochastic kinetics of ribosomes: single motor properties and collective behavior. Phys Rev E 80: 011908. 10.1103/PhysRevE.80.011908 19658730

[40] Dong JJ, Schmittmann B, Zia RKP (2007) Towards a model for protein production rates. Journal of Statistical Physics 128: 21–34.

[41] Brackley CA, Romano MC, Thiel M (2011) The dynamics of supply and demand in mRNA translation. PLoS Comput Biol 7: e1002203. 22022250 10.1371/journal.pcbi.1002203 PMC3192816

[42] Chou T (2003) Ribosome recycling, diffusion, and mRNA loop formation in translational regulation. Biophys J 85: 755–773. 12885626 10.1016/S0006-3495(03)74518-4 PMC1303200

[43] McCarthy JEG (1998) Posttranscriptional control of gene expression in yeast. Microbiology and Molecular Biology Reviews 62: 1492–1553. 9841679 10.1128/mmbr.62.4.1492-1553.1998 PMC98953

[44] Holstege FC, Jennings EG, Wyrick JJ, Lee TI, Hengartner CJ, et al (1998) Dissecting the regulatory circuitry of a eukaryotic genome. Cell 95: 717–728. 9845373 10.1016/s0092-8674(00)81641-4

[45] Warner JR (1999) The economics of ribosome biosynthesis in yeast. Trends in Biochemical Sciences 24: 437–440. 10542411 10.1016/s0968-0004(99)01460-7

[46] Frenkel-Morgenstern M, Danon T, Christian T, Igarashi T, Cohen L, et al (2012) Genes adopt non-optimal codon usage to generate cell cycle-dependent oscillations in protein levels. Molecular Systems Biology 8: 572. 22373820 10.1038/msb.2012.3 PMC3293633

[47] Ashe MP, De Long SK, Sachs AB (2000) Glucose depletion rapidly inhibits translation initiation in yeast. Molecular Biology of the Cell 11: 833–848. 10712503 10.1091/mbc.11.3.833 PMC14814

[48] Castelli LM, Lui J, Campbell SG, Rowe W, Zeef LAH, et al (2011) Glucose depletion inhibits translation initiation via eIF4A loss and subsequent 48S preinitiation complex accumulation, while the pentose phosphate pathway is coordinately up-regulated. Molecular Biology of the Cell 22: 3379–3393. 21795399 10.1091/mbc.E11-02-0153 PMC3172263

[49] Shenton D, Smirnova JB, Selley JN, Carroll K, Hubbard SJ, et al (2006) Global translational responses to oxidative stress impact upon multiple levels of protein synthesis. J Biol Chem 281: 29011–29021. 16849329 10.1074/jbc.M601545200

[50] Belle A, Tanay A, Bitincka L, Shamir R, O'Shea EK (2006) Quantification of protein half-lives in the budding yeast proteome. Proceedings of the National Academy of Sciences 103: 13004–13009. 10.1073/pnas.0605420103 PMC1550773 16916930

[51] Brockmann R, Beyer A, Heinisch JJ, Wilhelm T (2007) Posttranscriptional expression regulation: what determines translation rates? PLoS Computational Biology 3: e57. 17381238 10.1371/journal.pcbi.0030057 PMC1829480

