# Supplementary material for: Ribosome Traffic on mRNAs Maps to Gene Ontology: Genome-wide Quantification of Translation Initiation Rates and Polysome Size Regulation
Source: PLoS Comput Biol. 2013 Jan 31;9(1):e1002866. doi: 10.1371/journal.pcbi.1002866 (PMC3561044; doi:10.1371/journal.pcbi.1002866)
Supplement: Text S1 — Detailed description of the approaches used in the main text and regarding (1) the estimate of the hopping rates in the two-state model; (2) the method to classify sequences in abrupt, smooth and hybrid class; (3) other examples of randomisation (with constant amino-acid sequence) of codon arrangement and expected protein production rates (similar to Figure 6); (4) numerical details for the quantification of the initiation rates; (5) details on the computed energy of secondary structures ; (6) an accurate explanation of the alternative hypothesis for the ORF-length dependence of the initiation rate; (7) description of the computation of the p-values. (PDF) [file pcbi.1002866.s006.pdf]

**Supplementary Information:**

# Ribosome traffic on mRNAs maps to gene ontology: genome-wide quantification of translation initiation rates and polysome size regulation

Luca Ciandrini, Ian Stansfield, M. Carmen Romano

---

Contents


| | | |
|---|---|---|
| 1. | Estimation of hopping rates in the two-state model | 1 |
| 2. | Classification of sequences | 5 |
| 3. | Codon arrangement as a key determinant of protein production rate | 7 |
| 4. | Quantification of initiation rates | 7 |
| 4.1. | Pheromone treatment | 8 |
| 5. | Energy of secondary structures | 10 |
| 6. | ORF-length dependence of the initiation rate | 11 |
| 7. | Computation of p-values | 13 |
| 8. | Software and simulations outcome | 13 |
| | References | 14 |


---

## 1. Estimation of hopping rates in the two-state model

Assuming a spatially homogenous pool of tRNAs in the cell, the tRNA-capture rates $k_i$ are related to the inverse of the average arrival and recognition time of the cognate tRNA, and are determined as follows.

The total translation rate $\omega_i$ of codon $i$ is given by:

$$\frac{1}{k_i} + \frac{1}{\gamma} = \frac{1}{\omega_i}, \tag{1}$$

where $\gamma$ is the translocation rate, $\gamma = 35s^{-1}$ (see main text). An initial estimate of the hopping rates $k_j$ of the codons translated by the tRNA $j$ is

$$k_j = r\frac{GCN_j}{\sum_{j=1}^{41} GCN_j}, \tag{2}$$

where $GCN_j$ is the gene copy number of the tRNA of type $j$ with $j = 1, \ldots, 41$, and $r$ is a proportionality constant such that $\langle\omega_i\rangle = 10$ codons/s, $\langle\cdot\rangle$ representing the average value over all the codons. That means, codons that are translated by the same tRNA $j$ are initially assigned the same $k_i$ value. Therefore, effectively we would have only 41

different types of codons, corresponding to the 41 different types of tRNAs. However, experimental data suggest that the translation rates of codons using the G-U wobble are reduced by 39% compared to their G-C counterparts. Analogously, codons using the wobble I-C and codons using the wobble I-A are reduced by 36% relative to their I-U counterparts [1]. We reduce then the initially obtained rates accordingly. Furthermore, the average value of the total translation rate is calculated as it follows

$$\langle \omega_i \rangle = \sum_{i=1}^{61} \left( \frac{k_i \gamma}{k_i + \gamma} \right) \frac{n_i}{n}, \tag{3}$$

where $n_i$ is total number of codons in the cell of exactly type $i$, and $n = \sum_{i=1}^{61} n_i$. Equating this last equation to $10s^{-1}$, we obtain (numerically) the value for the proportionality constant $r$. Table S1 summarises the results. The termination rate is assumed to be fast and comparable to the translocation. Therefore, we set $\beta = 35s^{-1}$.

We are aware that additional factors can be taken into consideration to estimate the $k_i$ rates, for instance, the occupancy of the ribosomal A-site by other (non-cognate and near-cognate) tRNAs [2, 3]. However, the main factor determining the arrival of the cognate tRNA is its abundance [12], and the overall conclusions of this work hold even considering further corrections in the estimation of the $k_i$ rates. To demonstrate this beyond doubt, we compared the $k_i$ rates with alternative transition rates that consider the occupancy time of the ribosomal A-site by near- and non-cognate tRNAs. To produce those rates we used tRNA abundance data from [4] and assumed biochemical reaction rates measured *in vitro* and in particular circumstances [5], as these were the only available data. Figure S1 shows a comparison of both sets of $k_i$ rates, from which it is apparent that the relationship among different $k_i$ rates does not change much in the alternative set of values.

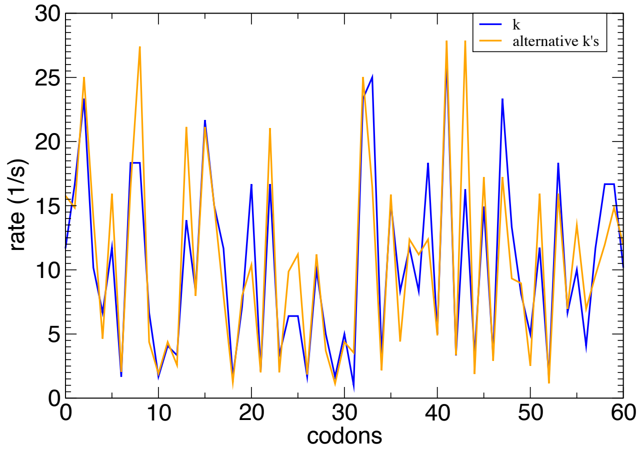


Figure S1. Comparison between the $k_i$ transition rates (blue) an alternative choice of the transition rates considering the competition of near- and non-cognate tRNAs (orange) for all codon types.

We additionally confirmed that our classification of sequences into two distinct types of response to initiation rate, namely *smooth* and *abrupt* (see main text), is not affected

by the additional consideration of non-cognate and near-cognate tRNAs. Moreover, as one can appreciate from Figure S2, the polysome size (density $\rho$) is comparable in both sets of rates, particularly around the expected value of the physiological initiation rate $\alpha_\varphi$ (which median is 0.09 $s^{-1}$ and mean 0.12 $s^{-1}$, see main text). For these reasons we did not to include this further level of complexity.

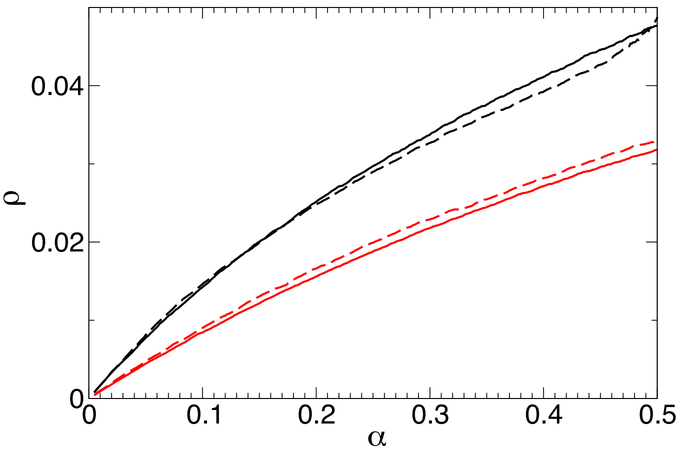


Figure S2. Comparison between the simulated densities computed by using the $k_i$ transition rates (full lines) and an alternative choice of the transition rates considering the competition of near- and non-cognate tRNAs (dashed lines) for two different mRNAs: YBL105C in black and YAL003W in red.

| tRNA | anti-codon | codon | GCN | $k_i$ |
|---|---|---|---|---|
| Ala1 | IGC | GCU | 11.00 | 18.34 |
| Ala1 | IGC | GCC | 11.00 | 11.74 |
| Ala2 | UGC | GCA | 5.00 | 8.34 |
| Ala2 | UGC | GCG | 5.00 | 8.34 |
| Arg1 | CCU | AGG | 1.00 | 1.67 |
| Arg2 | ICG | CGU | 6.00 | 10.01 |
| Arg2 | ICG | CGC | 6.00 | 6.40 |
| Arg2 | ICG | CGA | 6.00 | 6.40 |
| Arg3 | UCU | AGA | 11.00 | 18.34 |
| Arg4 | CCG | CGG | 1.00 | 1.67 |
| Asn | GUU | AAU | 10.00 | 10.17 |
| Asn | GUU | AAC | 10.00 | 16.68 |
| Asp | GUC | GAU | 15.00 | 15.26 |
| Asp | GUC | GAC | 15.00 | 25.01 |
| Cys | GCA | UGU | 4.00 | 4.07 |
| Cys | GCA | UGC | 4.00 | 6.67 |
| Gln1 | UUG | CAA | 9.00 | 15.01 |
| Gln2 | CUG | CAG | 1.00 | 1.67 |
| Glu3 | UUC | GAA | 14.00 | 23.35 |
| Glu4 | CUC | GAG | 2.00 | 3.34 |
| Gly1 | GCC | GGU | 16.00 | 16.28 |
| Gly1 | GCC | GGC | 16.00 | 26.68 |
| Gly2 | UCC | GGA | 3.00 | 5.00 |
| Gly3 | CCC | GGG | 2.00 | 3.34 |
| His | GUG | CAU | 7.00 | 7.12 |
| His | GUG | CAC | 7.00 | 11.67 |
| Ile1 | UAU | AUA | 2.00 | 3.34 |
| Ile2 | IAU | AUU | 13.00 | 21.68 |
| Ile2 | IAU | AUC | 13.00 | 13.87 |
| Leu1 | UAG | CUA | 3.00 | 5.00 |
| Leu1 | UAG | CUG | 3.00 | 5.00 |
| Leu3 | CAA | UUG | 10.00 | 16.68 |
| Leu4 | UAA | UUA | 7.00 | 11.67 |
| Leu5 | GAG | CUU | 1.00 | 1.02 |
| Leu5 | GAG | CUC | 1.00 | 1.67 |
| Lys1 | CUU | AAG | 14.00 | 23.35 |
| Lys2 | UUU | AAA | 7.00 | 11.67 |
| Met | CAU | AUG | 5.00 | 8.34 |
| Phe | GAA | UUU | 10.00 | 10.17 |
| Phe | GAA | UUC | 10.00 | 16.68 |
| Pro1 | UGG | CCA | 10.00 | 16.68 |
| Pro1 | UGG | CCG | 10.00 | 16.68 |
| Pro2 | IGG | CCU | 2.00 | 3.34 |
| Pro2 | IGG | CCC | 2.00 | 2.13 |
| Ser2 | IGA | UCU | 11.00 | 18.34 |
| Ser2 | IGA | UCC | 11.00 | 11.74 |
| Ser3 | GCU | AGU | 4.00 | 4.07 |
| Ser3 | GCU | AGC | 4.00 | 6.67 |
| Ser4 | UGA | UCA | 3.00 | 5.00 |
| Ser5 | CGA | UCG | 1.00 | 1.67 |
| Thr1 | IGU | ACU | 11.00 | 18.34 |
| Thr1 | IGU | ACC | 11.00 | 11.74 |
| Thr2 | CGU | ACG | 1.00 | 1.67 |
| Thr3 | UGU | ACA | 4.00 | 6.67 |
| Trp | CCA | UGG | 6.00 | 10.01 |
| Tyr | GIA | UAU | 8.00 | 8.14 |
| Tyr | GIA | UAC | 8.00 | 13.34 |
| Val1 | IAC | GUU | 14.00 | 23.35 |
| Val1 | IAC | GUC | 14.00 | 14.94 |
| Val2 | UAC | GUA | 2.00 | 3.34 |
| Val2b | CAC | GUG | 2.00 | 3.34 |

TABLE S1. Hopping rates $k_i$ considering supply (gene copy number of tRNAs) and wobble base-pairing.

## 2. Classification of sequences

In order to classify the mRNA sequences into *abrupt* or *smooth* we applied a clustering k-means based algorithm [17] to the density of particles in the excited state (ribosomes loaded with tRNAs before translocation to the next codon) versus the initiation rate $\alpha$. This curve can be approximated by three consecutive straight lines. In the case of *smooth* sequences, the slope of the first line is larger than the slope of the second line, whereas in the case of *abrupt* sequences it is the other way around. In the idealistic case of a sequence composed of only the same fast codon except a slow codon in the bulk of the sequence, the density of ribosomes shows a discontinuity when $\alpha$ reaches the value of the hopping rate of the slow codon [6]. A real mRNA sequence is composed of many different codons, but if there is a slow codon or a cluster of slow codons in the bulk of the sequence it can act as a bottleneck for the traffic of ribosomes. Due to the inhomogeneities of the sequence (every codon is different) and to the finite length of the sequence, there is no real discontinuity in the density of particles, but beyond a certain value of the initiation rate $\alpha$ the density increases rather abruptly, until it reaches the saturation value. Therefore, we find three qualitatively different regions in the curve; in the first region the density increases slowly with $\alpha$, in the second region the increase is very abrupt, and in the third region, the density shows a very shallow or no increase with $\alpha$ (see Figure S3b). In contrast, if the mRNA sequence does not contain any slow codons or cluster of slow codons in the bulk, but there are either no slow codons or they are at the beginning of the sequence, the density of particles increases smoothly with $\alpha$. In this case, the curve can also be split in three regions; in the first region the density of loaded ribosomes increases steadily with $\alpha$, in the second region the rate of increase decreases, until the saturation is reached in the third region (see Figure S3a).

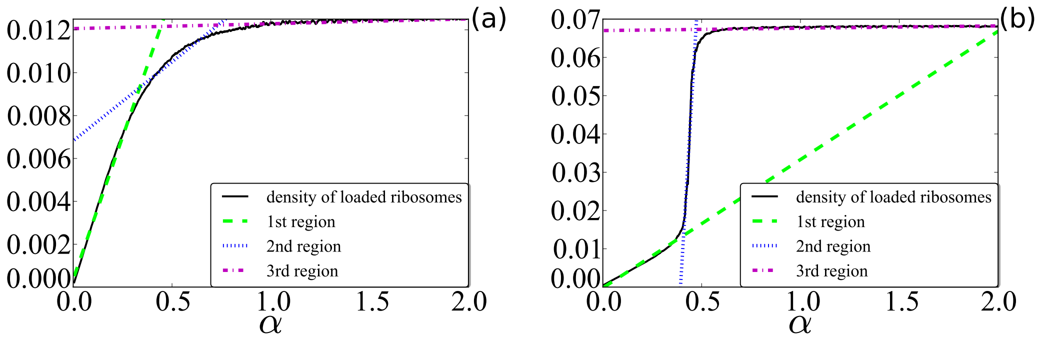


Figure S3. Slopes of the density of loaded ribosomes (ribosomes with a tRNA in their A site) in the three regions identified by the algorithm for a typical *smooth* sequence (YAL019W) (a) and a typical *abrupt* one (YAL001C) (b).

Hence, the k-means based algorithm iteratively assigns each point of the curve to one of the three regions, by performing a linear regression in each part. The iterative loop stops when there is no further change in the assignment of points to each of the three regions. By means of this algorithm we are able to classify 5,669 sequences out of all

6,293 ORFs. In the remaining sequences, the algorithm did not find three consecutive slopes to approximate the curve, but rather assigned different intermingled segments of the curve to the three different straight lines. In order to classify these sequences we applied another algorithm; we first performed a linear regression taking the first tenth of the points of the curve and kept adding consecutive points to the first region, every time updating the linear regression, until there was a large deviation from the initial slope. That point indicated the end of the first region of the curve. Then we continued with the third region of the curve, starting from the last point and going backwards. Like that, the second region, the most difficult to identify, was determined. We then calculated for each sequence a goodness of fit parameter between the simulated density curve and the fitted straight lines (normalised square root of the sum of squared differences) . In the first run of the second algorithm, the goodness of fit parameter for some sequences was too high (indicating a poor fit); hence, those sequences were reanalysed again by the second algorithm with changed parameters (the thresholds that limited the maximal allowed deviation from the initial first and third regions). Again, after this analysis some sequences presented a poor goodness of fit, and hence this procedure was reiterated 6 times. At the end, only 32 sequences could not be classified by means of these two algorithms.

In the final classification, we included an additional class for sequences on the boundaries between the two groups (for example, sequences initially classified as abrupt by the algorithms, but without pronounced traits of this class). Such sequences generally have an absolute relative difference between the slope of the second and the first part of the curve smaller than a pre-defined threshold, i.e. sequences with $|b_2 - b_1|/b_1 < 0.5$, where $b_1$ and $b_2$ denote the slopes of the first and second part of the curve, respectively. We denote these sequences as *hybrid*. The histogram of the abruptness parameter $(b_2 - b_1)/b_1$ for all sequences is presented in Fig. S4.

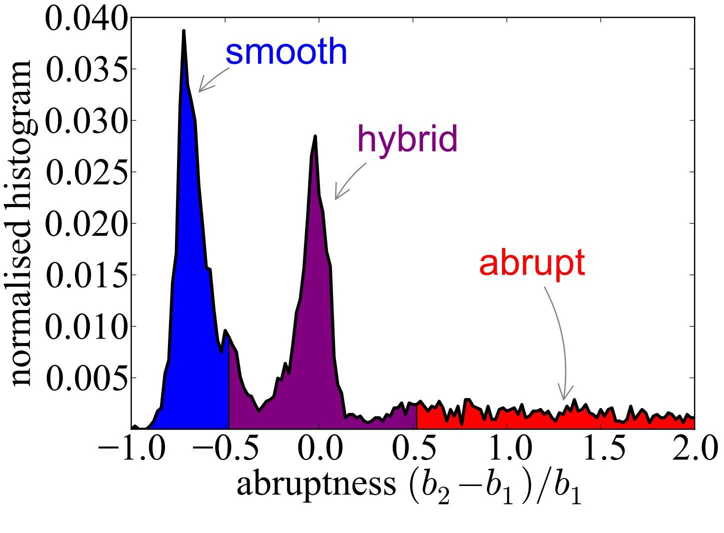


Figure S4. Normalised histogram of the abruptness $(b_2 - b_1)/b_1$ among the entire genome. Smooth sequences in blue (with abruptness $< 0.5$), abrupt sequences in red (abruptness $> 0.5$) and hybrid among them. The abruptness reaches values of $\sim 50$, but here we show only the first part of the distribution to emphasise the smooth and hybrid sequences.

## 3. Codon arrangement as a key determinant of protein production rate

To show that codon arrangement is a key determinant of protein production rate $J$ we randomised the codon configuration of a given mRNA in such a way that the codon composition is identical, as well as the encoded amino acid sequence. Applying this randomisation algorithm we generated 2,000 randomised surrogates of the original sequence and calculate the protein production rate $J$ for each one, for a fixed value of the initiation rate $\alpha$. We chose a large value of the initiation rate, namely $\alpha = 10\ s^{-1}$ in order to isolate the elongation effects.

The values of the obtained protein production rates are different among the randomised population and are collected in an histogram (an example is shown in Figure S5). Similar distributions can be obtained for smaller initiation rates.

The fact that we obtain a broad distribution of $J$ values clearly demonstrates that mRNAs with identical codon usage but different codon arrangement have different translation efficiency.

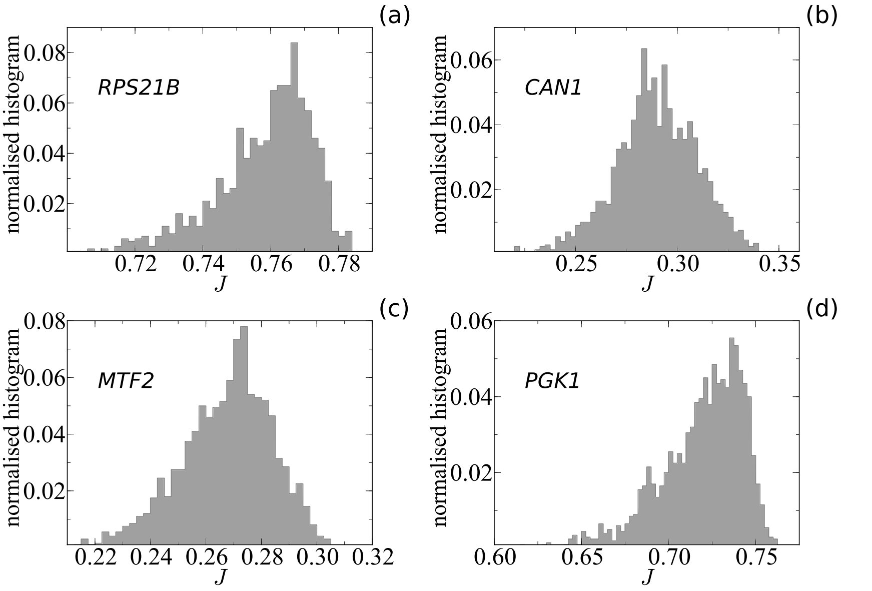


Figure S5. Normalised histograms of the simulated protein production rates $J$ obtained by randomising at least 1,000 times the sequences of the genes (a) *RPS21B* (YJL136C - ribosomal protein), (b) *CAN1* (YEL063C - membrane protein), (c) *PGK1* (YCR012W - phosphoglycerate kinase), and (d) *MTF2* (*YDL044C* - mitochondrial transcription factor).

## 4. Quantification of initiation rates

By combining the genome-wide experimental data of polysome size by [15] with the results from our stochastic simulations, we were able to extract the value of the initiation rate $\alpha$ for each mRNA. This was done by equating the measured (experimental) values and the outcome of the simulations, Equation (1) of the manuscript, via a linear

interpolation between the simulations points. The value of $\alpha$ was then determined as the value at which the simulated polysome size equated the experimental value.

We found a strong correlation between the ORF length and the estimated $\alpha_\varphi$ (see Figure 3 in the main text). In Fig. S6 we emphasise that, because each mRNA has a different rate of change of the density upon changes of the initiation rate (given by its codon composition and arrangement), an mRNA with high ribosome density does not necessarily mean that it has a large initiation rate. In the example shown, in fact, we illustrate how the estimated initiation rate of a gene with a large density value (YAL008W, in blue) is smaller than the initiation rate of a gene with smaller amount of ribosomes per nucleotide (YBL087C, in green).

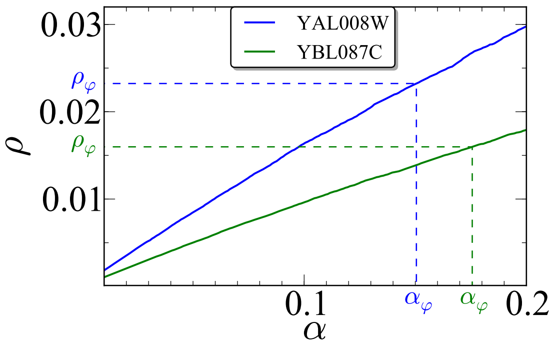


FIGURE S6. Procedure to estimate the physiological initiation rates $\alpha_\varphi$: the experimental [15] density $\rho_\varphi$ is used to extrapolate the physiological initiation rate $\alpha_\varphi$ for each transcript from the predicted ribosome densities (full lines).

We interpret the correlation between $\alpha_\varphi$ and the ORF length as the indication of a possible regulatory mechanism of the initiation of translation, likely due to the circularisation of smaller transcripts, that can control the recruitment and loading of ribosomes into the mRNAs, and lead to the known density-ORF length relationship [11] (see also Section 6 of this Supplementary Information).
Furthermore, to demonstrate that comparable results can be obtained by using an independent dataset, we used the density measurements from [11] and repeated the procedure obtaining a new set of initiation rate values denoted by $\alpha_\varphi^{Arava}$. We found again that the estimated values of the initiation rates are distributed rather than being a unique value for all the mRNAs, see Figure S7. The physiological $\alpha_\varphi$ used in the main text (where we used the dataset from [15]) and this set of initiation rates $\alpha_\varphi^{Arava}$ are highly correlated (Spearman's rank=0.73, p-value$< 10^{-6}$), confirming that our results are general and not only valid for a specific dataset.

4.1. **Pheromone treatment.** We used the same procedure to estimate the set of initiation rates $\alpha_t$ under pheromone treatment starting from the polysome measurements provided in [15]. We then compared the initiation rates under pheromone treatment to the physiological ones and defined, for each mRNA, the relative change as $(\alpha_\varphi - \alpha_t)/\alpha_\varphi$. Figure S8 shows the normalised histogram of the relative change across all genome.

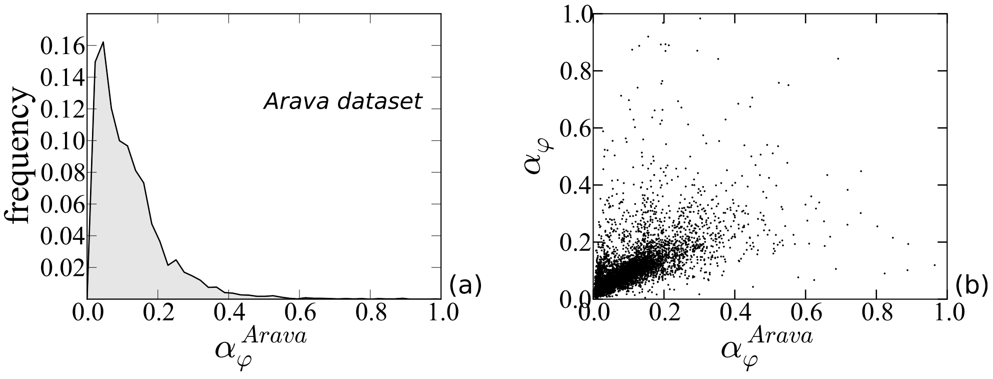


FIGURE S7. (a) Distribution of the estimated initiation rates in *S. cerevisiae* using polysome size measurements reported in [11]. (b) Comparison between the physiological initiation rates estimated using data of polysome size by [15] ($\alpha_\varphi$) and by [11] ($\alpha_\varphi^{Arava}$), Spearman's rank =0.73, p-value$< 10^{-6}$.

Although in general we do not detect large changes in the initiation process under pheromone treatment, two genes playing a key role in the pheromone response pathway (*SAG1* and *HO*) present a large variation in the initiation rate. Consistent with this observation, these two genes are known to be subject to alteration of their 5'UTRs under pheromone treatment [16].

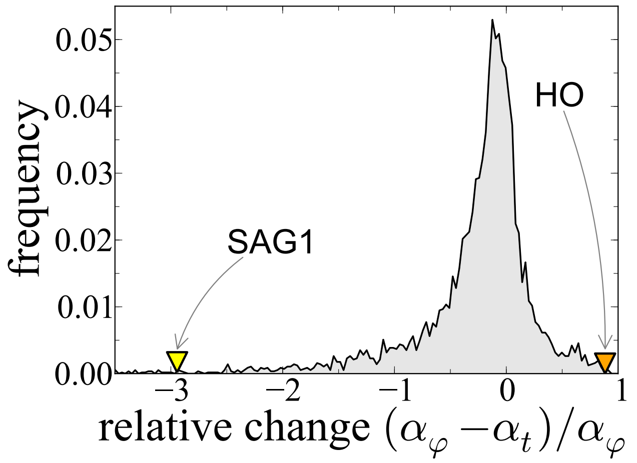


FIGURE S8. Frequency of the relative change of the initiation rates between the physiological and the pheromone cases, $(\alpha_\varphi - \alpha_t)/\alpha_\varphi$. Triangles indicate the corresponding values for *SAG1* and *HO*.

## 5. Energy of secondary structures

We computed the energy of secondary structures in the 5'UTRs (sequences from [7] with the RNAfold programme from the Vienna package [8]. The values of the free energies were then compared to the estimated initiation rates $\alpha_\varphi$; we found a significant correlation (Spearman's rank = 0.25, p-value$< 10^{-6}$) between the initiation rates and the free energy of secondary structures, meaning that folding structures in the 5' leaders can affect the recruitment of ribosomes. Figure S9 shows the scatter plot between the estimates $\alpha_\varphi$ and the absolute value of the free energy of secondary structures in the 5'UTRs.

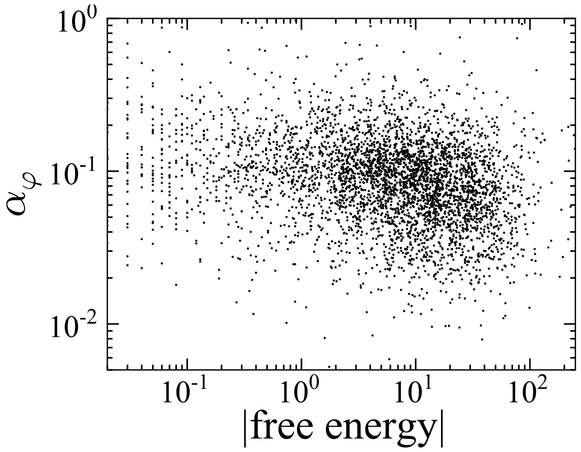


Figure S9. Scatter plot of the absolute value of the free energies of secondary structures in the 5'UTRs and the estimated $\alpha_\varphi$.

## 6. ORF-length dependence of the initiation rate

In [14] the authors argue that a region of constant length with higher ribosome density close to the 5' end (caused by the ramp of slow codons) would lead to the observed larger ribosome densities on short transcripts [11, 14], since that constant length high density region is a proportionately larger fraction of a small mRNA (see hypothesis A in Fig. S10). To test this argument, we have first determined the relative bottleneck position (position of the bottleneck divided by the length of the ORF) and we find, consistently with [13], slow regions at the start of the coding sequences (see Figure S11).

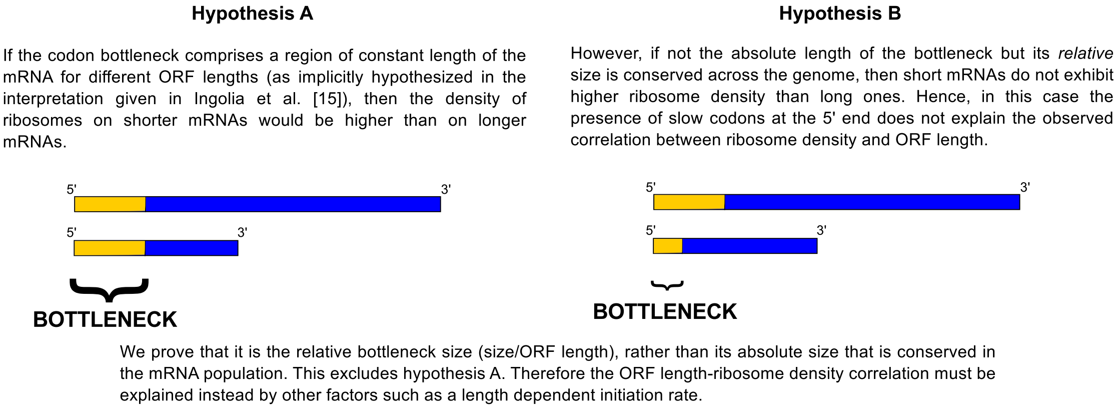


Figure S10. Two different hypotheses on the extension of the bottleneck close to the 5' end. (A) The bottlenecks has roughly the same extension independent on the ORF length. (B) The 5' bottleneck occupies a region which extension depends on the ORF length, and its relative length is similar for all the transcripts.

We computed the bottleneck position (using the $k_i$ rates, section 1 of this Supplementary Information) of each sequence of the genome, following the method described in [13]. We find that the ramp of slow codons at the beginning of the sequences does not have a fixed length (hypothesis A in Fig. S10), but it is shorter for shorter sequences, so that the relative position of the bottleneck to the total ORF length is constant across the genome (hypothesis B in Fig. S10). This is shown in the joint histogram of relative bottleneck position and ORF length in Figure S11; if hypothesis A were true, we would have observed the peak of the distribution moving towards 1 upon increments of the ORF length. The reader should notice that a similar conclusion was found by Noval and Pilpel [9].

The excess of ribosomes queueing at the 5' end would cause the density-ORF length correlation observed in the experiments [11, 14] only if hypothesis A were verified (same amount of ribosomes queueing would weight more for shorter mRNAs). Instead, in hypothesis B the amount of ribosomes queueing scales with the length of the transcript, and hence the excess of ribosomes at the 5' end cannot explain the experimental density-ORF length correlation.

We have also performed another test of hypothesis A, based on the stochastic dynamics of ribosomes; we compute the maximal density $\rho_{\max}$ as defined in the 'Materials and

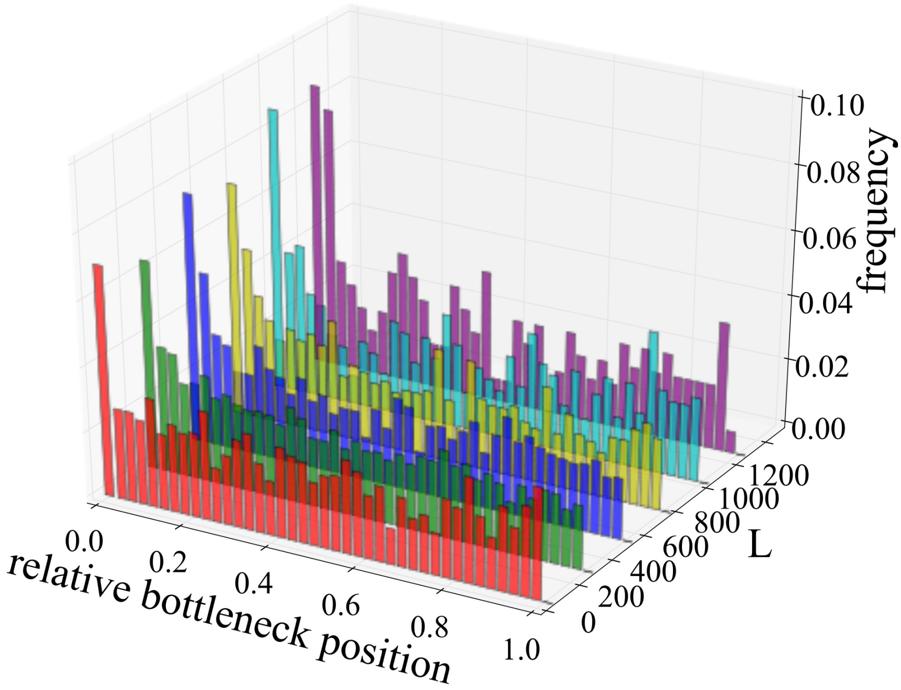


FIGURE S11. Joint histogram of the relative bottleneck position and length $L$ of the ORF. To compare different values of $L$ we normalise the marginal distributions of the relative positions (the sum of the frequencies of bins with the same $L$ is equal to 1). For any ORF length there is an accumulation of bottlenecks close to the 5' region (consistently with [13]). The relative position of the upstream bottlenecks rather than their absolute location, however, seems to be conserved.

Methods', which is determined by the relative position of the bottleneck, since after the bottleneck the density of ribosomes would be negligible. We find no significant correlation between ORF length and $\rho_{\max}$ (Figure S12), indicating that the position of the bottleneck does not depend on the transcript length; in other words, the average distance of the bottleneck from the beginning of the ORF is not constant across the genome.

Thus an elongation limited process (bottleneck) would not explain the negative correlation between densities and transcript lengths. We suggest instead, neglecting other possible sources of correlations such as mRNA degradation [10], that one possible reason for the negative correlation between coding sequence length and ribosome density is the relationship we detect here between initiation rates and ORF lengths. The negative correlation between initiation rates and ORF lengths may be a signature of ribosome recycling: due to the pseudo-circular conformation of mRNAs, the ends of short mRNA chains can relatively easily interact and enhance the ribosome re-initiation process, while

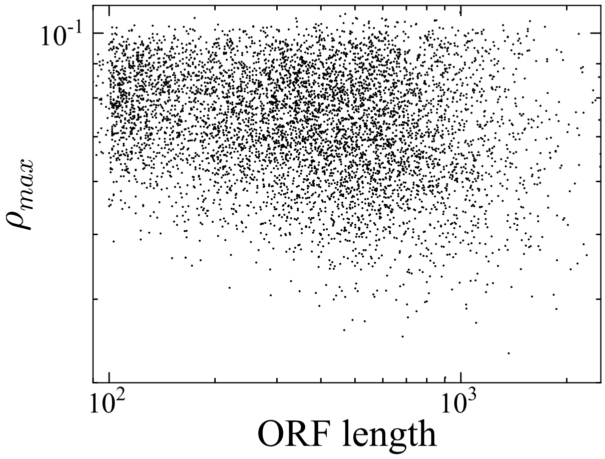


FIGURE S12. Scatter plot of ORF length against $\rho_{\max}$. Since $\rho_{\max}$ depends on the position of the bottleneck, the lack of clear correlation indicates that the bottleneck position cannot be the only cause of the experimentally observed density-length relationship.

long sequences are potentially less efficient recyclers of ribosomes [18, 19], as already speculated in [11].

## 7. Computation of p-values

The p-values given along with the correlation coefficients were calculated as follows: after computing the correlation coefficient $c(X,Y)$ between the data sets $X = \{x_i\}$ and $Y = \{y_i\}$, one of the data sets, say $Y = \{y_i\}$, was shuffled $10^6$ times, thereby generating $10^6$ surrogates $Y_j^{\text{surr}}$, with $j = 1, \ldots, 10^6$. Then, the correlation coefficient was computed between $X$ and each of the generated surrogates, leading to a set of $10^6$ correlation coefficients values $c(X, Y_j^{\text{surr}})$. These values were then ranked, and the proportion of these values above $c(X,Y)$ determined the p-value.

To avoid confusion, the measure of statistical significance provided by an hypergeometric function is instead denoted with a capital letter, P-value.

## 8. Software and simulations outcome

The programs used were written in C++ and are available on request. For all mRNAs, the profiles of density and current for different values of initiation rates, $\rho(\alpha)$ and $J(\alpha)$, are also available on request or can be downloaded from the authors' webpages or from http://abdn.ac.uk/mRNA-translation/.

## References

[1] Gilchrist MA, Wagner A (2006) A model of protein translation including codon bias, nonsense errors, and ribosome recycling. J Theor Biol 239: 417434.

[2] Fluitt A, Pienaar E, Viljoen H (2007) Ribosome kinetics and aa-tRNA competition determine rate and fidelity of peptide synthesis. Comput Biol Chem 31: 335346.

[3] Chu D, Barnes DJ, von der Haar T (2011) The role of tRNA and ribosome competition in coupling the expression of different mRNAs in saccharomyces cerevisiae. Nucleic Acids Research .

[4] Varenne S, Lazdunski C (1986) Effect of distribution of unfavourable codons on the maximum rate of gene expression by an heterologous organism. J Theor Biol 11: 99–110.

[5] Rodnina MV, Wintermeyer W (2001) Fidelity of aminoacyl-tRNA selection on the ribosome: kinetic and structural mechanisms. Annual Review of Biochemistry 70: 415–435.

[6] Kolomeisky AB (1998) Asymmetric simple exclusion model with local inhomogeneity. Journal of Physics A 31: 1153–1164.

[7] Nagalakshmi U, Wang Z, Waern K, Shou C, Raha D, et al. (2008) The transcriptional landscape of the yeast genome defined by RNA sequencing. Science 320: 1344 –1349.

[8] Hofacker IL, Fontana W, Stadler PF, Bonhoeffer LS, Tacker M, et al. (1994) Fast folding and comparison of RNA secondary structures. Monatshefte fur Chemie Chemical Monthly 125: 167–188.

[9] Navon S, Pilpel Y (2011)The role of codon selection in regulation of translation efficiency deduced from synthetic libraries. Genome Biology 12:R12.

[10] Valleriani A, Zhang G, Nagar A, Ignatova Z, Lipowsky R (2011) Length-dependent translation of messenger rna by ribosomes. Phys Rev E 83: 042903.

[11] Arava Y, Wang Y, Storey JD, Liu CL, Brown PO, et al. (2003) Genome-wide analysis of mRNA translation profiles in saccharomyces cerevisiae. Proc Natl Acad Sci U S A 100: 38893894.

[12] Gingold H, Pilpel Y (2011) Determinants of translation efficiency and accuracy. Mol Syst Biol 7.

[13] Tuller T, Carmi A, Vestsigian K, Navon S, Dorfan Y, et al. (2010) An evolutionarily conserved mechanism for controlling the efficiency of protein translation. Cell 141: 344–354.

[14] Ingolia NT, Ghaemmaghami S, Newman JRS, Weissman JS (2009) Genome-wide analysis in vivo of translation with nucleotide resolution using ribosome profiling. Science 324: 218–23.

[15] MacKay VL, Li X, Flory MR, Turcott E, Law GL, et al. (2004) Gene expression analyzed by high-resolution state array analysis and quantitative proteomics. Molecular & Cellular Proteomics 3: 478 –489.

[16] Law GL, Bickel KS, MacKay VL, Morris DR (2005) The undertranslated transcriptome reveals widespread translational silencing by alternative 5' transcript leaders. Genome Biology 6: R111.

[17] Press W, Teukolsky S, Vetterling W, Flannery B (2007) Numerical Recipes 3rd Edition: The Art of Scientific Computing. Cambridge Univ Press.

[18] Chou T (2003) Ribosome recycling, diffusion, and mRNA loop formation in translational regulation. Biophys J 85: 755773.

[19] McCarthy JEG (1998) Posttranscriptional control of gene expression in yeast. Microbiology and Molecular Biology Reviews 62: 1492 –1553.
