# Supplementary material for: Ribosome Traffic on mRNAs Maps to Gene Ontology: Genome-wide Quantification of Translation Initiation Rates and Polysome Size Regulation
Source: PLoS Comput Biol. 2013 Jan 31;9(1):e1002866. doi: 10.1371/journal.pcbi.1002866 (PMC3561044; doi:10.1371/journal.pcbi.1002866)
Supplement: Text S2 — Comparison with the previous model by Siwiak and Zielenkiewicz [22]. (PDF) [file pcbi.1002866.s007.pdf]

**Supplementary Information:**

# Ribosome traffic on mRNAs maps to gene ontology: genome-wide quantification of translation initiation rates and polysome size regulation

Luca Ciandrini, Ian Stansfield, M. Carmen Romano

---

## 1. Comparison with other models

In contrast to other approaches (e.g. by Siwiak and Zielenkiewicz [4]) that do not consider ribosome dynamics but assume an homogeneous occupancy of the transcript by ribosomes, i.e. a flat density profile, our model explicitly accounts for the complex movement and residence times of the ribosomes on the different codons.

With our present understanding of the translation process, for example thanks to the studies by Tuller and coworkers [1, 3] and with the experimental observation by Ingolia et al. [2], nowadays one can hardly neglect the importance of the steric interaction between ribosomes, particularly next to the 5' end of the transcript.

Without considering the interaction between ribosomes, the polysome size (and thus the ribosome density) would increase linearly with the initiation rate; our method instead estimates the physiological initiation rates $\alpha_\varphi$ starting from the numerical, mRNA-dependent, relations $\rho(\alpha)$, which are not linear and allow us to differentiate the transcript in different classes (*smooth*, *abrupt*, and *hybrid*).

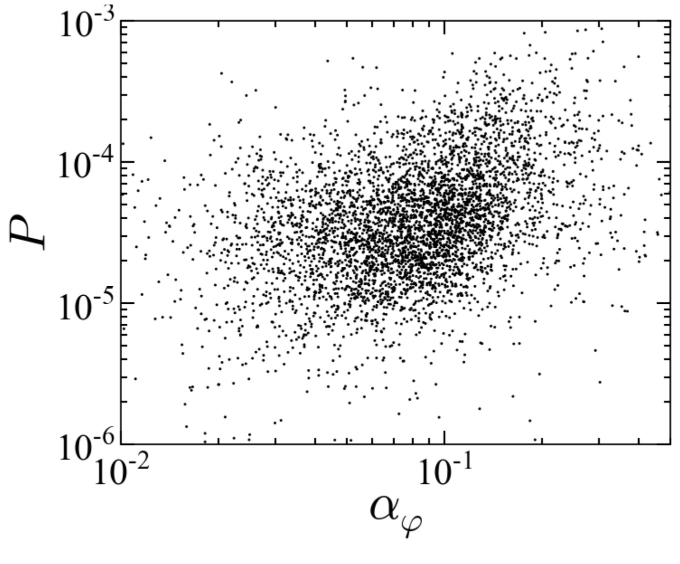


Figure S1. Comparison between the estimated physiological initiation rates $\alpha_\varphi$ (in $s^{-1}$) and the equivalent quantity $P$ found in [4].

For all these reasons, we would expect relevant changes in the initiation rates estimated with our approach or with more simple methods that do not examine the role of ribosome traffic. To show that, we have produced a scatter plot between the estimates $\alpha_\varphi$ and the initiation rates $P$ from [4]. The results, presented in Fig. S1, show that the values are only poorly correlated (Pearson $= 0.25$, p-value $< 10^{-6}$), meaning that by considering the ribosome dynamics we can capture important additional information neglected in previous approaches.

## References

[1] Tuller T, Carmi A, Vestsigian K, Navon S, Dorfan Y, et al. (2010) An evolutionarily conserved mechanism for controlling the efficiency of protein translation. Cell 141: 344–354.

[2] Ingolia NT, Ghaemmaghami S, Newman JRS, Weissman JS (2009) Genome-wide analysis in vivo of translation with nucleotide resolution using ribosome profiling. Science 324: 218–23.

[3] Reuveni S, Meilijson I, Kupiec M, Ruppin E, Tuller T (2011) Genome-Scale analysis of translation elongation with a ribosome flow model. PLoS Comput Biol 7: e1002127.

[4] Siwiak M, Zielenkiewicz P (2010) A Comprehensive, Quantitative, and Genome-Wide Model of Translation. PLoS Comput Biol 6(7): e1000865.
